# Chemoinformatic Characterization of NAPROC-13: A Database for Natural Product ^13^C NMR Dereplication

**DOI:** 10.1021/acs.jnatprod.4c00530

**Published:** 2024-09-13

**Authors:** Juan F. Avellaneda-Tamayo, Naicolette A. Agudo-Muñoz, Javier E. Sánchez-Galán, José L. López-Pérez, José L. Medina-Franco

**Affiliations:** † DIFACQUIM Research Group, Department of Pharmacy, School of Chemistry, Universidad Nacional Autónoma de México, Avenida Universidad 3000, Mexico City 04510, Mexico; ‡ Science and Technology Faculty, Universidad Tecnológica de Panamá, Campus Metropolitano Víctor Levi Sasso, Avenida Universidad Tecnológica, Vía Puente Centenario, Panama City 0819-07289, Panama; § Facultad de Ingeniería de Sistemas Computacionales, Universidad Tecnológica de Panamá, Campus Metropolitano Víctor Levi Sasso, Avenida Universidad Tecnológica, Vía Puente Centenario, Panama City 0819-07289, Panama; ∥ Grupo de Investigación en Biotecnología, Bioinformática y Biología de Sistemas (GIBBS), Universidad Tecnológica de Panama, Panama City, Panama; ⊥ Departamento de Ciencias Farmacéuticas, Área de Química Farmacéutica, Facultad de Farmacia, CIETUS, IBSAL, Campus Miguel de Unamuno, University of Salamanca, 37007, Salamanca, Spain; # Departamento de Farmacología, Facultad de Medicina, CIPFAR, Universidad de Panamá, Panama City, Panama

## Abstract

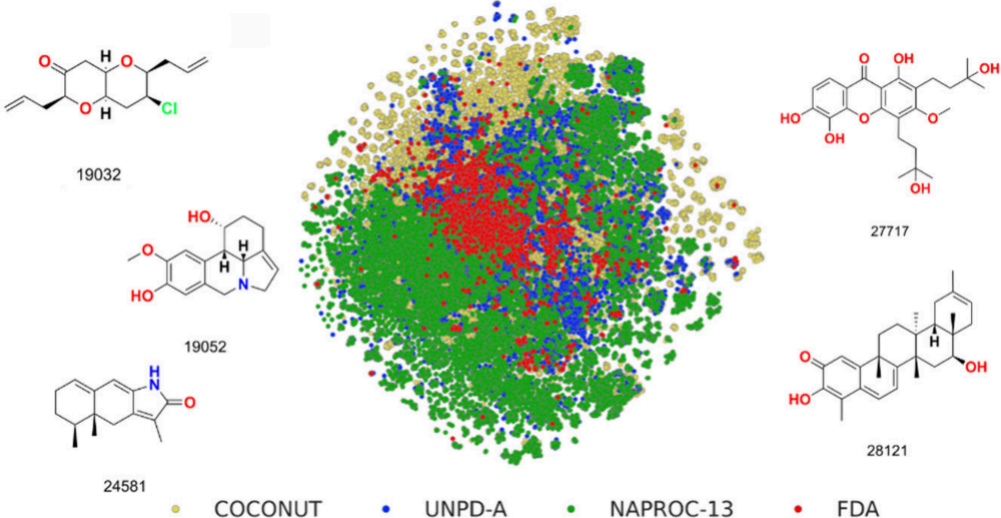

Natural products (NPs) are secondary metabolites of natural origin with broad applications across various human activities, particularly the discovery of bioactive compounds. Structural elucidation of new NPs entails significant cost and effort. On the other hand, the dereplication of known compounds is crucial for the early exclusion of irrelevant compounds in contemporary pharmaceutical research. NAPROC-13 stands out as a publicly accessible database, providing structural and ^13^C NMR spectroscopic information for over 25 000 compounds, rendering it a pivotal resource in natural product (NP) research, favoring open science. This study seeks to quantitatively analyze the chemical content, structural diversity, and chemical space coverage of NPs within NAPROC-13, compared to FDA-approved drugs and a very diverse subset of NPs, UNPD-A. Findings indicated that NPs in NAPROC-13 exhibit properties comparable to those in UNPD-A, albeit showcasing a notably diverse array of structural content, scaffolds, ring systems of pharmaceutical interest, and molecular fragments. NAPROC-13 covers a specific region of the chemical multiverse (a generalization of the chemical space from different chemical representations) regarding physicochemical properties and a region as broad as UNPD-A in terms of the structural features represented by fingerprints.

Natural products (NPs) are chemical compounds produced by organic life to carry out secondary metabolic processes. NPs and their derivatives are a well-known source of bioactive compounds, as well as widely applied compounds in many other areas of human life.^1^ The importance of discovering, characterizing, and developing the area of NPs lies in their broad spectrum of molecular complexity and structural diversity, as well as those known as privileged scaffolds, because of their optimization through evolution over millions of years.^2−4^ Noteworthy, more than 66% of current drugs approved for clinical use are NPs or NP derivatives.^5^ The size of the currently explored chemical space of NPs comprises more than 1 × 10^6^ compounds, both isolated and predicted.^6^ Different estimates indicate that between 2.5 × 10^5^ and 4 × 10^5^ are available in public compound databases.^7,8^ The number of newly reported molecules keeps increasing.^9,10^ Because of these reasons, NPs continue to be of interest to chemists and specialists of all disciplines regarding the possibility of present privileged properties in terms of material resistance,^11^ biological activity in drug discovery,^1,10,12^ fertilizer industry,^13^ and pesticides.^14^ NPs present an advantage, as bioactive candidates, over synthetic compounds, because they are synthesized by living species and have been exploited traditionally by different cultures at different stages of history.^15^

As part of the NP-based drug discovery protocols, it is crucial to establish whether the bioactive compound from natural sources is a new finding. To this purpose, chromatograms–spectra of hit mixtures are compared to those of reference using multiple powerful data analysis tools to identify potential bioactive compounds. This approach is the basis of natural product (NP) dereplication and takes advantage of metabolomics and other omics technologies.^16^

Structural elucidation and dereplication of NPs are rapidly advancing fields that benefit greatly from data analysis and machine learning. These technologies enable the efficient processing of large volumes of data generated from various experimental methods, which are becoming increasingly sensitive and high-resolution. As a result, researchers face more complex challenges in analyzing diverse chemical systems and handling vast amounts of data. Machine learning helps overcome these challenges by identifying patterns and relationships in large data sets, ultimately improving the speed and accuracy of NP identification and characterization.^17^

Data related to NPs, and specifically to NP dereplication, are stored in databases. Existent NP databases in the public domain have been reviewed recently by Sorokina and Steinbeck, until 2020.^18^ Previously, in 2015, Johnson and Lange reviewed open-access metabolomics databases for NP research.^19^ Databases focused on NP dereplication relate structural information to spectral and/or chromatographic information on compounds and taxonomic knowledge on NP sources.^17^ Those types of databases can be classified according to the spectrometric or spectroscopic information they support, i.e., MS or NMR data.

NAPROC-13 is a freely web-based accessible and searchable database at https://c13.materia-medica.net/, that collects structural and ^13^C NMR spectral information on NPs, of which a considerable number of their structures have been reviewed.^20−23^ NAPROC-13 contains information regarding 24 722 compounds (21 250 after applying a chemoinformatics-based curation protocol), mostly from plant sources, and in less amount from marine and microbiological organisms. By geographical distribution, systematic introductions of NPs are limited to those from Panama and El Salvador. Most of the entries in NAPROC-13 come from various unexplicit countries worldwide. Information on the chemical structures is not freely downloadable. NAPROC-13 has captured more relevance each year as evidenced by the 106 citations, 619 downloads, and 2916 views of the original publication (updated to May 1, 2024)^24^ and has more than 800 registered users from many countries around the world. In addition, a recent publication reporting on the usefulness of NAPROC-13 as a new strategy for finding erroneously established NPs contains reports of over 3200 views.^25^

The main goal of this study is to analyze quantitatively the chemical content, diversity, and coverage in the chemical space of NPs in the latest release of NAPROC-13. Chemoinformatic and statistical tools were applied for this purpose. The database was compared to the Food and Drug Administration of the United States (FDA)-approved drugs and a diverse subset of NPs (known as UNPD-A)^2^ regarding physicochemical and constitutional descriptors. To this end, we computed molecular descriptors of pharmaceutical interest, among others, and discussed the structural complexity as quantified by standard metrics. Also, we contrasted the structural diversity of compounds in NAPROC-13 according to different types of representations: (1) molecular fingerprints of different designs, (2) scaffold content according to Bemis and Murko’s definition,^26^ (3) ring systems, and (4) computed list of fragments using the RECAP algorithm of fragmentation.^27^ The ring systems of the studied data sets were correlated versus a virtual database of ring systems with reported bioactivity, Magic Rings, computed and released by Ertl.^28^

Also, we analyzed the chemical multiverse of NAPROC-13 using different types of molecular representations, including physicochemical, constitutional, and fingerprint-based descriptors. The chemical multiverse is a set of multiple chemical spaces, describing the same set of molecules through different sets of descriptors, each one describing the compounds differently but in a complementary manner.^29^ We used the COlleCtion of Open Natural ProdUcTs (COCONUT) database to approach the known chemical space of NPs. Results of this investigation quantitatively showed the large structural diversity of NPs. The results also confirmed the vast chemical diversity of the NAPROC-13 database, which is well suited for NP dereplication and virtual screening on different target families, including the report of more than 4000 new NPs. Some examples of those new included natural products are shown in Figure 1. NAPROC-13 showed a high structural diversity as well as the presence of interesting substructures (molecular scaffolds, Magic Rings, and molecular fragments) suitable for the discovery and design of drug candidates, including the development of pseudo-NPs.^30^

**Figure 1:**
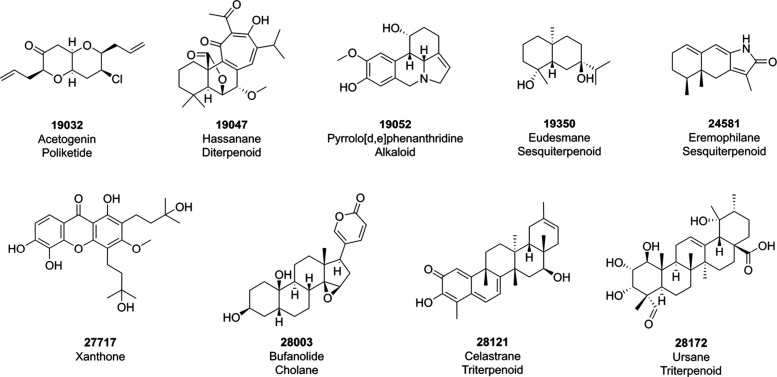
Examples of nine new molecules (from more than 4000) reported in NAPROC-13 that are not present in the current version of COCONUT, with their configuration. NAPROC-13 ID, NP type, and group are underneath each structure (19047 and 19052 correspond to the revised structures from the original publications).

## Results and Discussion

### Data Sets

Information in Table 1 summarizes relevant data in the analysis of NAPROC-13 and the reference data sets studied in the present work. The number of exclusive entries refers to the number of compounds that do not share among the other databases. The SSE refers to the entropy of the information (compounds) distributed among the number of different possible values (scaffolds) presented in the particular data set. The molecular similarity is a measure of the diversity of compounds within each database, in this case, according to their ECFP4 representation. CSP3 and chiral centers are different estimations of molecular complexity.^31,32^ The NPL score was computed according to the methods section.

**Table 1:** Structural Constitution, Complexity, and NPL Score of NAPROC-13 and Reference Data Sets

data set	size (initial)	size (curated)	exclusive entries (%)^b^	Murcko scaffolds (SSE)	mean (median) similarity ECFP4 1024 bits	mean (median) CSP3	mean (median) chiral centers	mean (median) NPL score
NAPROC-13	24722	21250	19992	0.97	0.144 (0.135)	0.668 (0.724)	6.586 (6.0)	2.437 (2.575)
FDA	2587	2324	2215	0.63	0.096 (0.094)	0.454 (0.429)	2.305 (1.0)	1.513 (1.505)
UNPD-A^a^	14994	14994	13676	0.67	0.099 (0.091)	0.519 (0.522)	3.806 (2.0)	0.019 (−0.095)

a Data set curated from the source.^2^

b Computed disregarding chirality of compounds.

Finally, 4327 NPs in NAPROC-13 were unique when compared against the current version of COCONUT.^8^ This finding reveals the valuable work of correcting elucidated NP structures. Some of their structures and classifications are represented in Figure 1.

### Distribution of Physicochemical Properties, Constitutional Descriptors, and Structural Complexity

**Figure 2:**
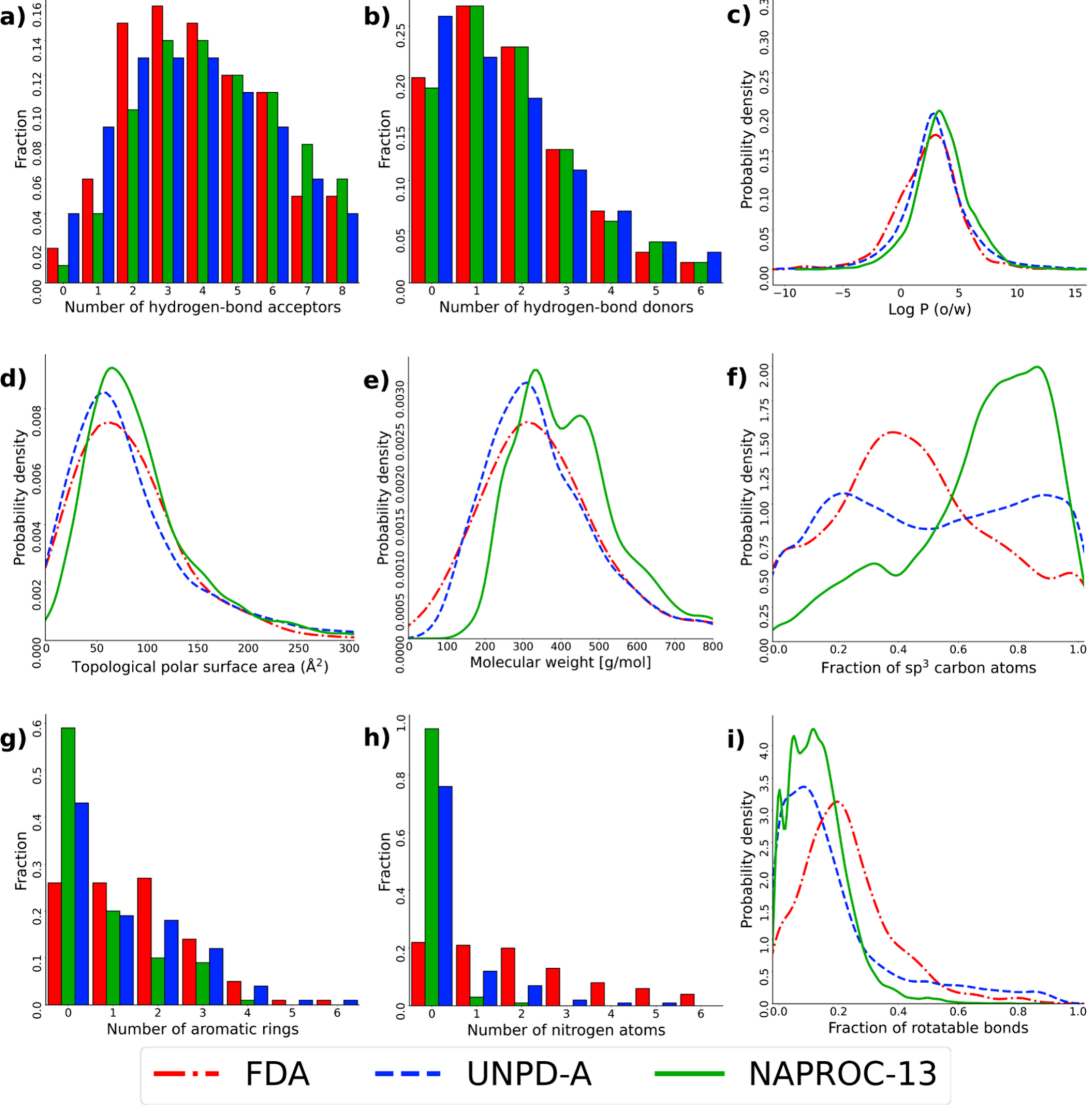
Distribution of physicochemical properties and constitutional descriptors among NAPROC-13 (green), FDA-approved drugs (red), and UNPD-A (blue): (a) HBA, (b) HBD, (c) logP, (d) TPSA, (e) MW, (f) CSP3, (g) number of aromatic rings, (h) number of nitrogen atoms, and (i) fraction of RB. Dotted lines are used for ease of visualization.

Figure 2 shows the distribution of molecular descriptors computed, both physicochemical properties and constitutional descriptors of interest in NPs and pharmaceutical research. Histograms represent the fraction of compounds sharing the referenced property within the database, while probability distributions depict the probability density functions for the variable. The importance of measuring properties such as those described by Lipinski^33^ and Veber^34^ lies in empirical rules developed in observing trends in most of the small molecules approved for clinical use that are administered orally. NPs in NAPROC-13 had on average 6.07 HBA (median: 5.00; standard deviation: 4.2), FDA-approved drugs had 5.29 (median: 4.00; standard deviation: 4.6), and NPs in UNPD-A had 5.58 (median: 4.00; standard deviation: 5.0). For HBD, NPs in NAPROC-13 had 2.27 on average (median: 2.00; standard deviation: 2.4), FDA-approved drugs 2.45 (median: 2.00; standard deviation: 3.7), and NPs in UNPD-A 2.51 (median: 2.00; standard deviation: 3.2). In this sense, the number of heteroatoms per molecule follows a similar trend. NPs in NAPROC-13 presented 6.25 on average (median: 5.00; standard deviation: 4.2), FDA-approved drugs 7.50 (median: 6.00; standard deviation: 7.0), and NPs in UNPD-A 6.02 (median: 5.00; standard deviation: 5.1).

The content of atoms distinct from carbon and hydrogen, which is directly correlated with the polarity of molecules, further influences properties like LogP and TPSA. Regarding LogP, NPs in NAPROC-13 presented an average of 3.54 (median: 3.48; standard deviation: 2.4), whereas FDA-approved drugs presented an average of 2.27 (median: 2.55; standard deviation: 2.9) and NPs in UNPD-A 2.94 (median: 2.87; standard deviation: 3.0). For TPSA, NPs in NAPROC-13 presented an average of 96.13 Å^2^ (median: 80.92 Å^2^; standard deviation: 64.1 Å^2^), FDA-approved drugs 95.72 Å^2^ (median: 74.60 Å^2^; standard deviation: 106.3 Å^2^), and NPs in UNPD-A 90.78 Å^2^ (median: 69.67 Å^2^; standard deviation: 82.7 Å^2^).

The molecular size was approximated by calculating the MW. NPs in NAPROC-13 presented an average of 430.38 g/mol (median: 404.46 g/mol; standard deviation: 163.6 g/mol), FDA-approved drugs had 387.38 g/mol (median: 337.37 g/mol; standard deviation: 272.0 g/mol), and NPs in UNPD-A 371.94 g/mol (median: 330.29 g/mol; standard deviation: 196.4 g/mol). The trend of bigger molecules is explainable due to large triterpenoids, which is the most frequent molecular class, and their functionalities, sometimes sugars. There is a scarcity of nitrogen atoms in NPs of NAPROC-13, which lies in an average of 0.06 per molecule (median: 0.00; standard deviation: 0.3), while FDA-approved drugs have 2.54 per molecule (median: 2.00; standard deviation: 3.3), and NPs in UNPD-A have 0.49 per molecule (median: 0.00; standard deviation: 1.2).

In general, the values of the descriptors discussed so far highlight that about 75% of the NPs in NAPROC-13 are within the values of the classical empirical drug-likeness rules, except for MW, with a Q3 of 502.52 (exceeding the rule by less than 1%).

### Molecular Complexity

Molecular complexity is a useful property in screening compounds against biological targets and indicates the selectivity in the interaction.^35^ Herein we discuss the complexity of studied databases according to topological indexes such as CSP3 and the number of chiral centers, and by the substructure-based approaches of ring quantification.^35,36^ NPs in NAPROC-13 presented an average CSP3 of 0.67 (median: 0.724; standard deviation: 0.2), FDA-approved drugs of 0.45 (median: 0.43; standard deviation: 0.3), and NPs in UNPD-A of 0.52 (median: 0.52; standard deviation: 0.3). According to chiral centers, NPs in NAPROC-13 had an average of 6.59 (median: 6.00; standard deviation: 5.1), FDA-approved drugs 2.31 (median: 1.00; standard deviation: 3.8), and NPs in UNPD-A 3.81 (median: 2.00; standard deviation: 5.1). In terms of ring content, molecules of NAPROC-13 had an average of 3.92 (median: 4.00; standard deviation: 1.72), FDA-approved drugs 2.78 (median: 3.00; standard deviation: 2.0), and NPs in UNPD-A 3.10 (median: 3.00; standard deviation: 2.2). These values reinforce the tendency of NPs to have greater complexity according to the metrics implemented. Also, a higher complexity of NPs in NAPROC-13 versus those in UNPD-A is observed. Previous findings show that a higher molecular complexity affords a better performance in clinical trials,^35^ in conjunction with better bioavailability-related physicochemical properties.^37^ These observations suggest a high potential in NAPROC-13 as a database suitable for virtual screening and lead development and optimization. A comprehensive visualization of molecular descriptors computed in this study and their descriptive statistical summary can be found in the Supporting Information, in Figure S2 and Table S1, respectively.

### Solubility-Descriptors Correlation

NAPROC-13 includes information about deuterated solvents utilized for solubilizing each compound during ^13^C NMR experimental analysis. In this study, we propose leveraging this data set as an indicator of NP solubility. Deuterated chloroform was the most frequently reported solvent, utilized for 13 481 compounds, followed by pyridine-*d*_5_ (2496 compounds), methanol-*d*_4_ (2060 compounds), acetone-*d*_6_ (822 compounds), dimethyl sulfoxide-*d*_6_ (DMSO-*d*_6_, 1415 compounds), and benzene-*d*_6_ (309 compounds). This solvent data can serve as a valuable component of a classification model, as is or with the inclusion of broader data. Relevant potential applications of this database are the prediction of ^13^C NMR signals and the impact of the analysis solvent as well as the putative development of specific solubility drugs of natural origin.

While the development of a thoroughly validated machine learning model itself falls beyond the scope of this study, the exploratory data analysis presented herein showed the potential of the data annotated in NAPROC-13 for predicting the solubility of NPs. A key methodology employed to assess the impact of various descriptors on solubility involves visualizing data on the compounds under study. This statistical approach enhances our understanding of the relationships between different properties and solubility categories, thereby facilitating the identification of interdependent variables and refining parameters that are crucial for training machine learning models. Moreover, this data set is potentially useful to train a predictive machine learning model in the field of the NMR signal analysis of NPs.

**Figure 3:**
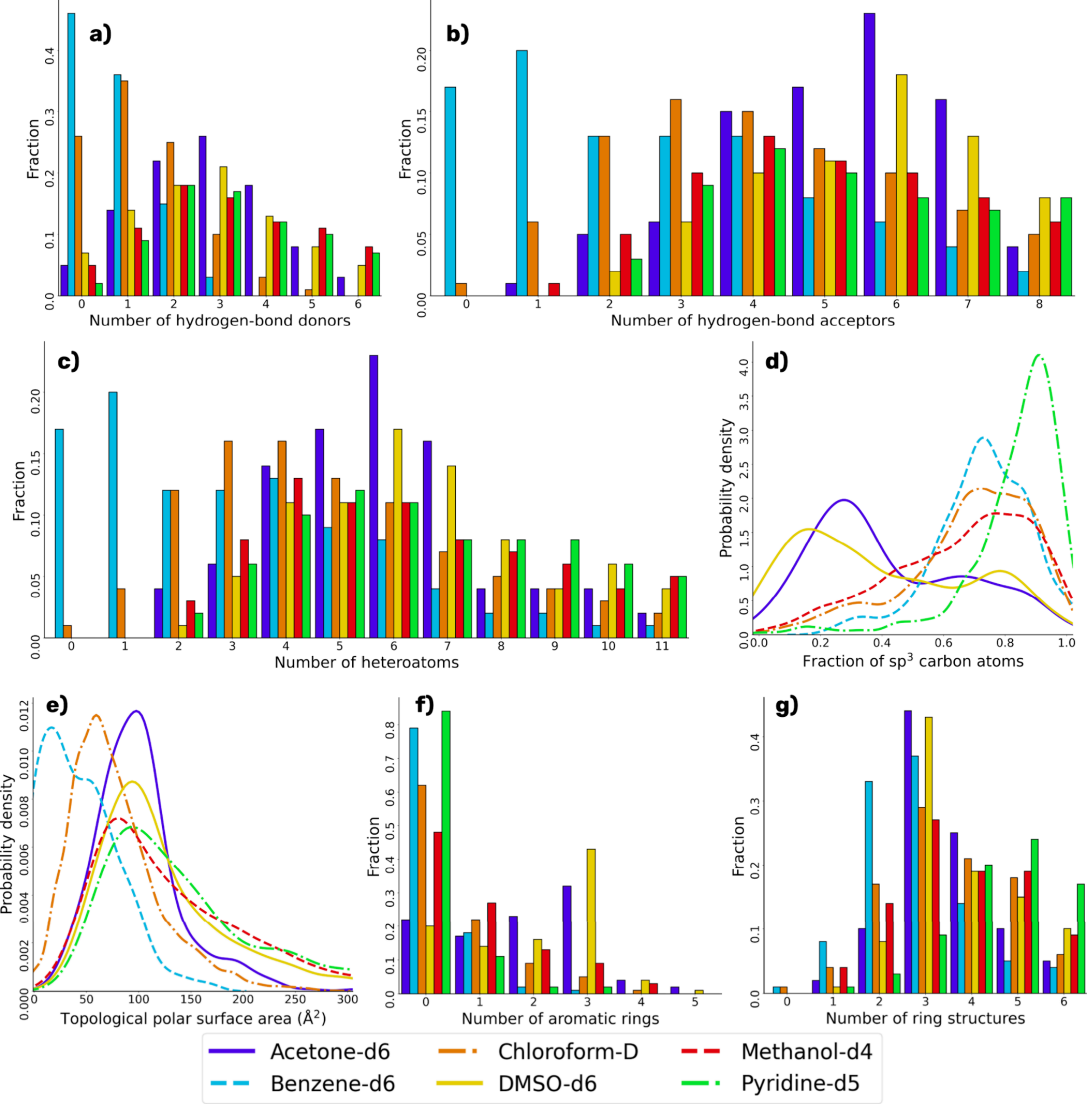
Distribution of physicochemical properties and constitutional descriptors among NAPROC-13 solubility categories. (a) HBD, (b) HBA, (c) number of heteroatoms, (d) CSP3, (e) TPSA, (f) number of aromatic rings, and (g) number of ring structures. Dotted lines are used for ease of visualization.

Figure 3 shows the distribution of selected molecular descriptors among the different categories of solubilities for NPs in NAPROC-13. Of note, Figure 3 is focused on NAPROC-13 compounds, while Figure 2 compares the profile of different descriptors of NAPROC-13 as compared to reference data sets. A complete visualization of molecular descriptors and a statistical descriptive summary are shown in Figure S3 and Table S2 in the Supporting Information, respectively. In Figure 3, the differential distribution of properties among the categories of analysis solvent (approximable to solubility) is included in NAPROC-13. Solubility is a macroscopic property of molecules in the presence of third substances, commonly associated with physicochemical/constitutional molecular properties, similar to the melting and boiling points. Some of the striking differences in this respect are related to the polarity and possibility of interacting through hydrogen bonds of molecules and to the polar/nonpolar character of the solvent. For example, some of these interesting relationships are the differential distribution of HBD and HBA, the number of heteroatoms, and CSP3.

### Structural Diversity

#### Fingerprint-Based Structural Diversity

**Figure 4:**
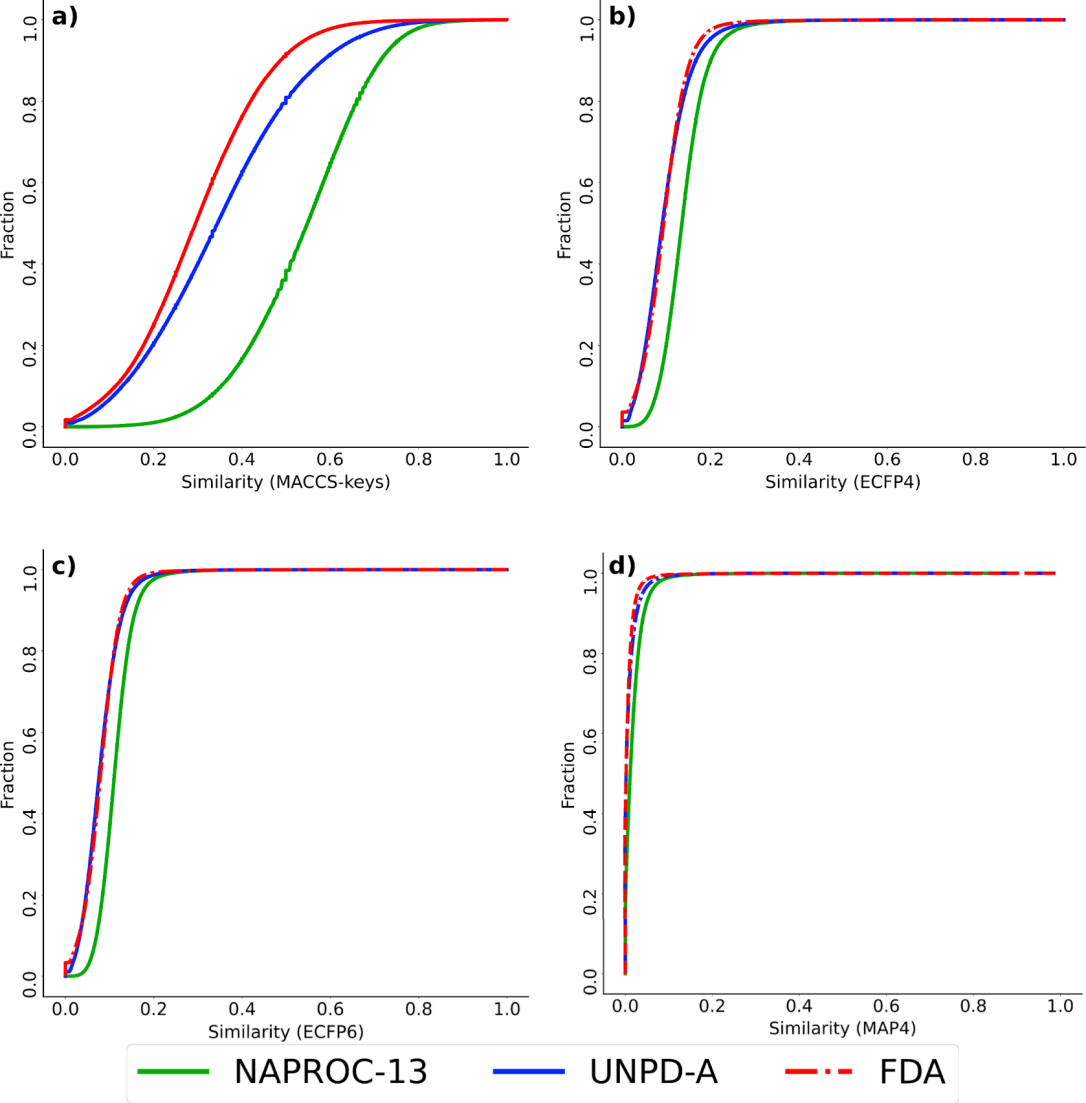
Cumulative distribution functions for the pairwise Tanimoto similarity using (a) MACCS-keys (166 bits), (b) ECFP4, (c) ECFP6, and (d) MAP4 fingerprints as molecular representations. NAPROC-13 (green), FDA-approved drugs (red), and the most diverse subset of NPs (UNPD-A, blue). Dotted lines are used for ease of visualization.

The structural fingerprints of different designs served as molecular representations across the studied data sets, with the Tanimoto similarity coefficient employed to gauge pairwise similarity between molecules. To assess diversity within each database, pairwise molecular similarity was computed, ascendingly ordered, and depicted as cumulative curves. A higher squared curve signifies greater database diversity, as illustrated in Figure 4. Independent of the molecular representation method used—whether MACCS keys (166 bits), ECFP4 or ECFP6 (1024 bits), or the recently developed MAP4 (2048 bits)—database diversity can be ranked in ascending order as follows: NAPROC-13 exhibits a lower diversity than UNPD-A, which, in turn, is less diverse than FDA-approved drugs. Statistical similarity values also indicate a lower diversity within compounds in NAPROC-13. However, it is important to highlight that even without this aim, NAPROC-13 demonstrates a diversity close to that of UNPD-A, despite the latter being designed to be highly diverse.^2^ NAPROC-13 closely aligns with the statistical similarity values of UNPD-A, particularly when employing molecularly independent fingerprints such as ECFP and MAP4. These descriptors offer higher resolution in molecular representation, detailing specific connectivity features along each molecule, thereby contributing to their effectiveness in assessing molecular diversity.^38,39^

### Scaffold-Based Substructural Diversity

**Figure 5:**
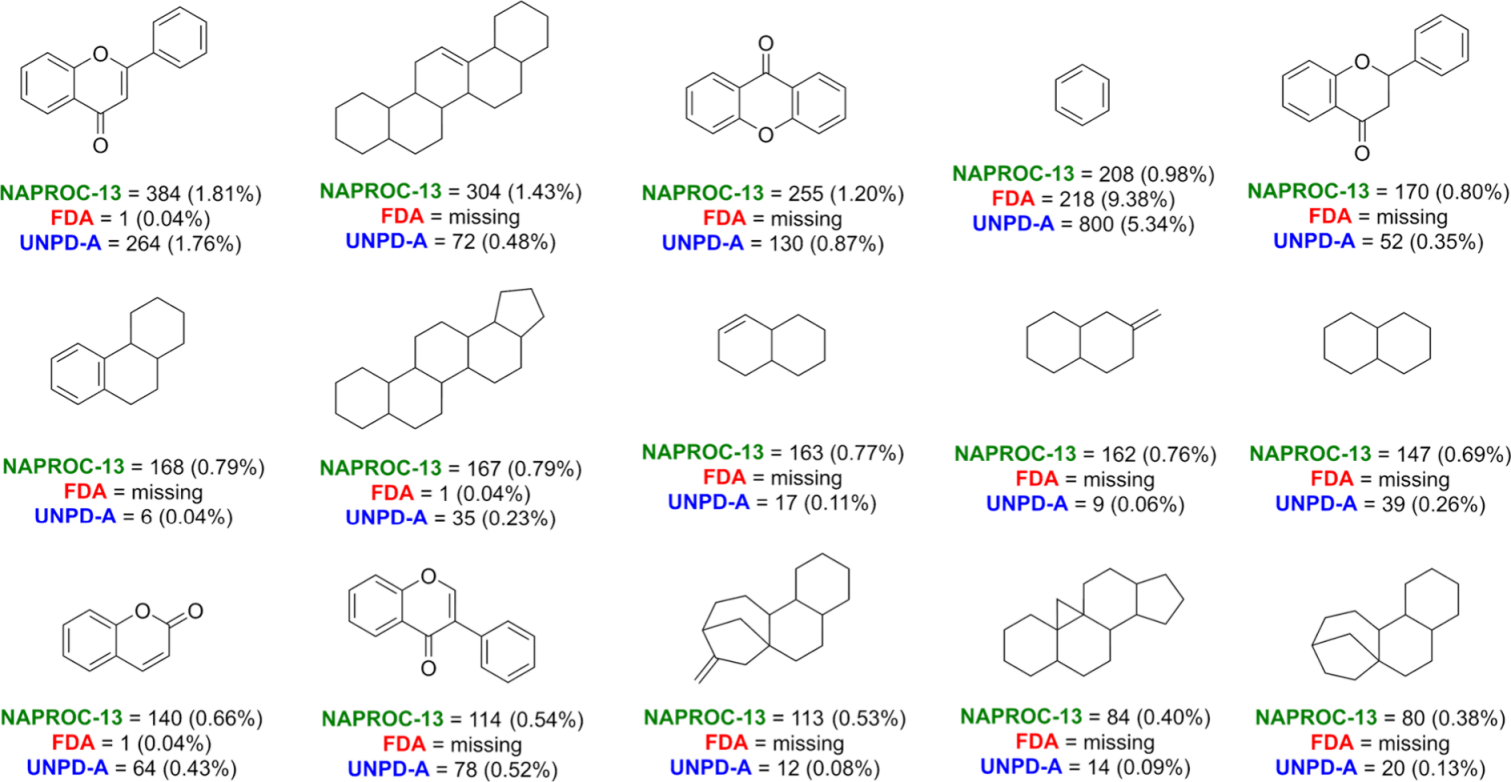
Fifteen of the most frequent scaffolds from NAPROC-13 and their abundances in the three data sets. Additionally, 165 (0.78%) of NAPROC-13, 265 (11.40%) approved drugs, and 1743 (11.62%) of UNPD-A are acyclic compounds.

Analysis of the scaffold content of the compound data sets revealed that compounds in NAPROC-13 present a high content of cyclic compounds, with a small fraction (0.78%) of acyclic structures. In contrast, NPs in UNPD-A, as well as FDA-approved drugs, have a similar fraction of acyclic compounds, nearly 11.5% of the database. However, it is remarkable that most of the cyclic molecular cores in NAPROC-13 are uniquely present in NAPROC-13 (Figure 5 and Figure S1 in the Supporting Information). Moreover, most of them are absent in the compounds approved for clinical use.

The large abundance of merged aromatic and saturated rings for almost all of the 15 most frequent scaffolds in NAPROC-13 is noticeable. An exception to this trend is the high content of benzene-type rings, with 208 (0.98%) substructures, which tend to be the most frequent scaffold in small-molecule databases,^40^ specifically in NPs, and even food chemicals.^4,26,39,41^

NAPROC-13 presented 8169 molecular scaffolds, 77 common to all three databases, 1770 shared with UNPD-A (the highest overlapping in scaffold analysis), and 84 shared with FDA-approved drugs. UNPD-A had 7059 scaffolds, 5175 were unique, and 191 were shared with FDA-approved drugs. Finally, FDA-approved drugs had 1291 scaffolds, and 1093 of them were unique. These results are in accordance with the high molecular diversity in NPs,^42^ demonstrated by pairwise similarity relationships (see above), and with previous analyses of the databases analyzed in this study.^2,39^

Quantitative approaches to compare the scaffold diversity of compound data sets are the SSE and the CSR curve. The scaled SSE is an index that measures how the information, in this case, compounds, is distributed along the different scaffolds, in terms of uniformity. The SSE ranges from zero (no diversity since all compounds share the same scaffold) to one (maximum diversity, uniform distribution of compounds along the scaffolds). For the three databases compared in the present study, NAPROC-13 presented an SSE of 0.97 for their 15 most frequent scaffolds (chemical structures shown in Figure 5), FDA-approved drugs 0.63, and UNPD-A 0.67. These values of SSE indicate that there is no evident predominance among the first 15 scaffolds in NAPROC-13 NPs. In contrast, for UNPD-A NPs as well as FDA-approved drugs, saturated and unsaturated six-membered rings are overrepresented, which can be noticeable in SSE. These results are in agreement with previous studies.^39^

**Figure 6:**
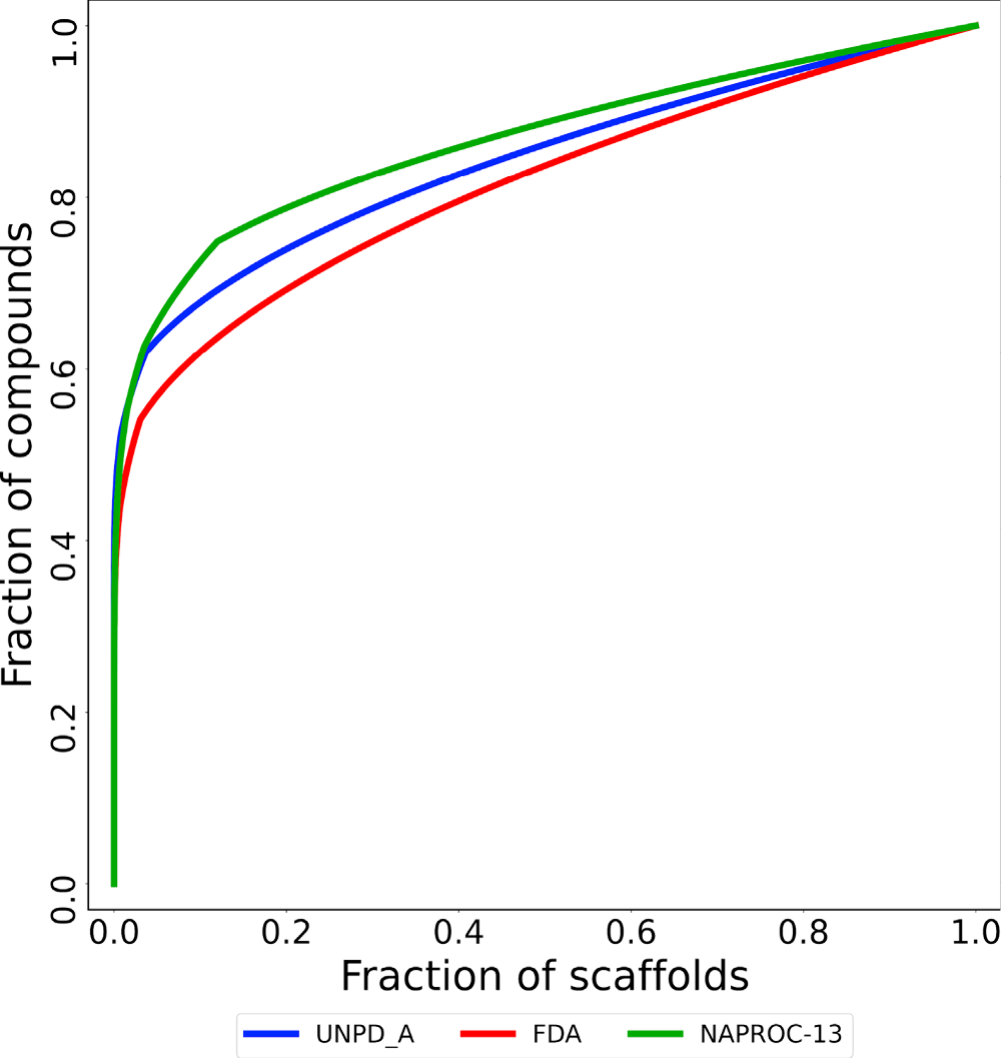
Cyclic system retrieval (CSR) curves for NAPROC-13 (green), FDA-approved drugs (red), and the most diverse subset of NPs (UNPD-A, blue).

The CSR curves shown in Figure 6 are a graphic representation of how compounds, represented as the fraction of each database, are distributed along the set of scaffolds, represented as the fraction of the total amount of scaffolds. As more compounds are accumulated by a small fraction of the database’s scaffolds, the less diverse the database is. The CSR curves in Figure 6 indicate that the FDA-approved drugs set has the overall largest scaffold diversity, followed by UNPD-A and finally by NAPROC-13. These measures support the previously discussed high diversity of the NPs.

To further compare the substructures in the different data sets, we analyzed the types and frequency of disconnected ring systems, which allows us to analyze the overlap sharing of minimal cyclic substructures among the three databases. The results of this analysis are reported in the next section.

### Ring System-Based Substructural Diversity

A ring system is defined as a set of atoms contained within a cycle including exocyclic double bonds. Unlike Bemis and Murcko scaffolds, ring systems disconnect the not-ring cores bonded by single bonds. This approach of molecular exploration has been widely implemented to describe the structural composition of bioactive molecules^43−47^ and has been used in the study of NPs.^48^ Possible combinations of heteroatom-containing ring systems have also been predicted, with a probable prediction of an activity for a biological target, under the categories of active, inactive, or undefined. Herein, we used the results reported by Ertl to extract and compare potential biologically active ring systems (so-called “Magic Rings”) and search for similarities among NAPROC-13 and reference databases.^28^ Ertl categorization classifies a ring system for a preferred family target if the report includes at least twice as large as the next target class; on the other hand, it is assigned to the multitarget category. In the category of bioactivity, a ring system is reported as active if it possesses at least ten times more reports as active than inactive. The same condition applies to the inactive classification. Otherwise, it does not assign a classification.^28^ Herein, we use the “intermediate” bioactivity class for such molecules.

In the current analysis, we identified 8902 ring systems; 3853 were only present in NAPROC-13 NPs, 2973 were only present in UNPD-A, and 438 were only present in FDA-approved drugs. The major overlapping set was between NAPROC-13 and UNPD-A, as expected, with 1504 ring systems. Between UNPD-A and FDA-approved drugs, there is an overlap of 229 ring systems and between NAPROC-13 and FDA-approved drugs there is an overlap of 109 ring systems. Finally, 102 ring systems are shared among the three databases (see Figure S1.C in the Supporting Information).

The main classes of bioactive ring systems identified in the three databases according to Ertl′s categorization, with classification either active or intermediate active, are related to multitarget reports (not-known: NAPROC-13: 728, FDA-approved drugs: 33, UNPD-A: 570; multiple targets: NAPROC-13: 153, FDA-approved drugs: 147, UNPD-A: 241; other enzymes: NAPROC-13: 143, FDA-approved drugs: 73, UNPD-A: 186). However, GPCR (NAPROC-13: 54, FDA-approved drugs: 76, and UNPD-A: 99) and nuclear receptors (NAPROC-13: 21, FDA-approved drugs: 10, UNPD-A: 32) are also well represented by natural product ring systems, both in NAPROC-13 and UNPD-A (see Figure S4 in the Supporting Information).

**Figure 7:**
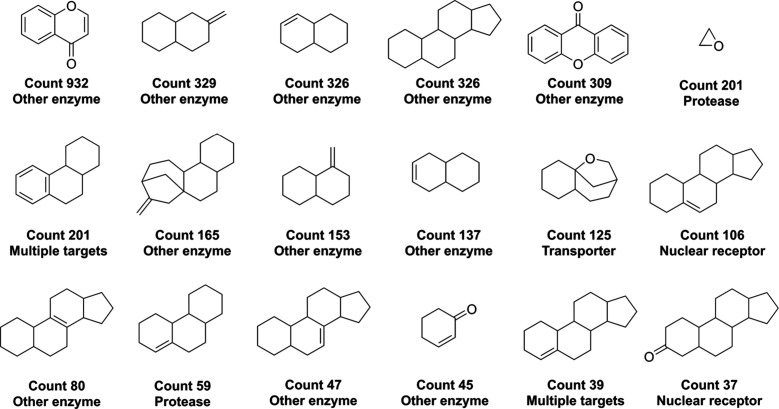
Most frequent potentially bioactive ring systems in NAPROC-13 (“Magic Rings”).^28^

Figure 7 shows the most frequent active ring systems (“Magic Rings”) in NAPROC-13 NPs. Figures S5 and S6 in the Supporting Information show the most frequent active ring systems in FDA-approved drugs and UNPD-A NPs, respectively.

The predominant ring system in the NP databases under study is the chromone (1-benzopyran-4-one) core, which serves as the structural foundation for flavonoids. Flavonoids, abundant in plants, play pivotal roles in essential metabolic pathways and have been linked to various biological activities, notably their antioxidant properties.^49,50^ Remarkably, this ring system is also prevalent among FDA-approved drug structures. Additionally, both NP databases exhibit a significant presence of diverse steroid-type cores, which are well-documented for their wide-ranging bioactivities.^51^

In their most frequent ring systems, UNPD-A exhibits nitrogen-containing ring systems in limited quantities, while NAPROC-13 lacks such structures entirely. Contrastingly, both databases include oxygen-containing ring systems. These characteristics align with the typical patterns observed in natural products and contrast with synthetic compounds like FDA-approved drugs.^10,52,53^

Conversely, UNPD-A displays a marked abundance of quinone-type cores, a feature notably absent in NAPROC-13. Quinones are intriguing NPs known for their dual nature, possessing both beneficial and potentially toxic effects in humans. This characteristic positions quinones as a promising area for ongoing and future investigations into their therapeutic potential and safety profile.^54^

### Fragment-Based Substructural Diversity

Molecular fragments are relevant entities for a comprehensive characterization of chemical libraries, specifically for drug design. Fragment-based drug design (FBDD) is founded in the search for better atomic efficiency in binding interaction.^55^ Those can be applied in the de novo design,^56^ reaction-based,^57^ or rules of transformation-based^58^ fragment-based libraries. In the field of fragment screening, more than 20 years ago Congreve et al. proposed a set of empirical rules known as the rule of three (RO3, MW < 300, cLogP ≤ 3, HBD ≤ 3, HBA ≤ 3).^59^ Although the RO3 has limitations, the concept has given relevant resources in FBDD, especially limiting the molecular complexity of fragments libraries, giving place to an adequate exploration of diverse zones of the molecular space and avoiding “molecular obesity”.^60−62^

Currently, there are multiple commercial or free accessible fragment libraries, many of them based on small synthetically accessible molecules, focused on particular biological activities, among others. However, there is still a lack of NP-based fragment libraries.^2,63^ For this reason, we consider that a highly valuable contribution of the present study is to make freely available the fragment libraries we have built in the Supporting Information.

We identified 748 533 different fragments, 405 265 were only present in NAPROC-13 NPs, 320 143 were unique in UNPD-A, and 14 141 were only present in FDA-approved drugs. The highest overlap was between both natural source databases with 8438 fragments in common. FDA-approved drugs and NAPROC-13 shared 345 fragments, UNPD-A and FDA-approved drugs 745, and 272 fragments were present in the three databases (see Figure S1.D in the Supporting Information).

**Figure 8:**
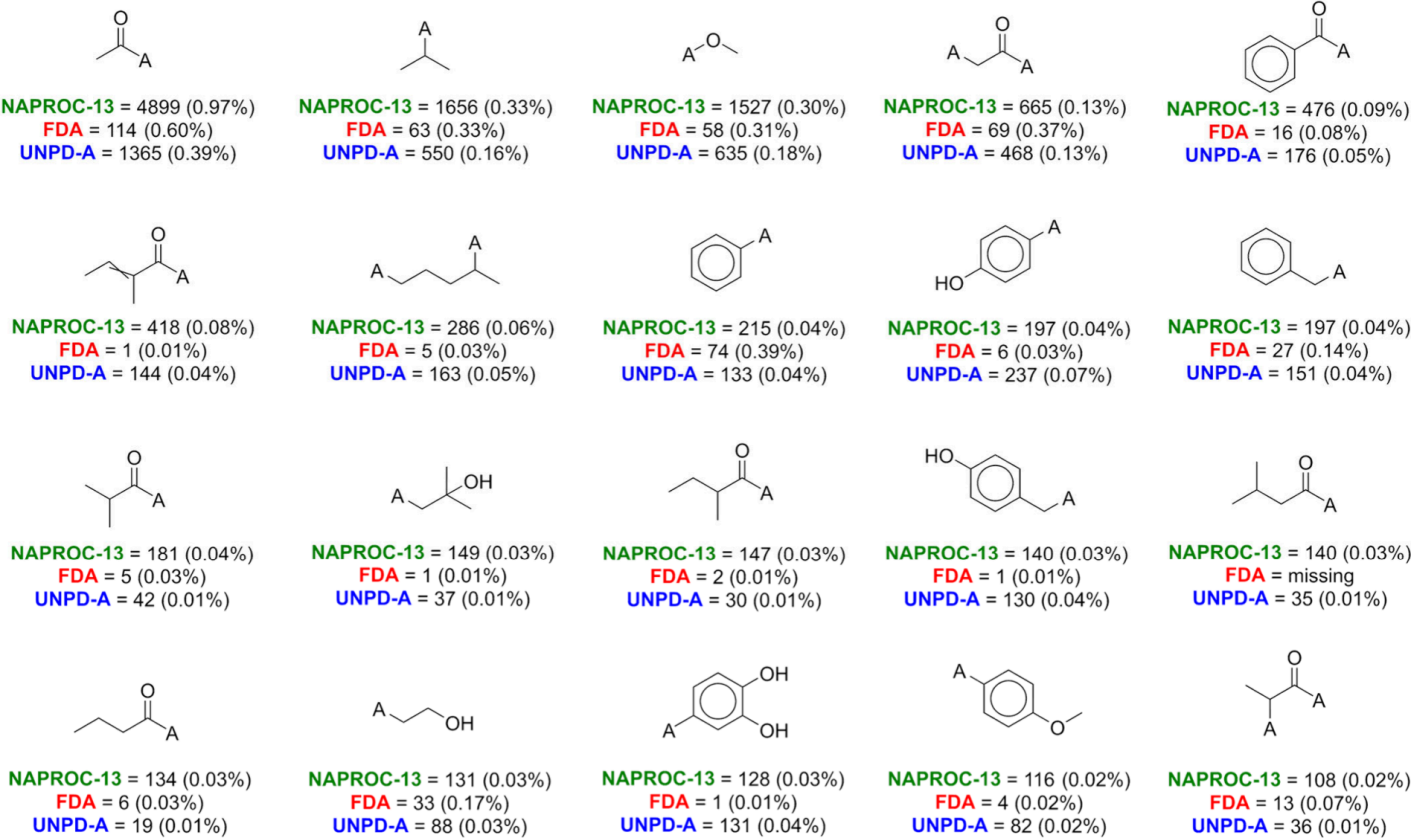
Most frequent fragments complaining of RO3 computed for NPs in NAPROC-13, and their frequencies in FDA-approved drugs and UNPD-A. “A” symbol means the point of disconnection.

For each data set of molecular fragments generated, we computed the properties included in the RO3. Figure 8 shows the most frequent molecular fragments in NAPROC-13 NPs fulfilling the RO3 conditions and their frequencies in FDA-approved drugs and NPs in UNPD-A.

Different molecular fragments fulfilling the RO3 conditions were 5196 for NAPROC-13 NPs, 2911 for FDA-approved drugs, and 7596 for UNPD-A NPs, highlighting the highest diversity of the UNPD-A subset. The computed fragments for the NP source databases presented a higher content of oxygen atoms in accordance with previous studies. Lack of heteroatoms more than oxygen is a characteristic mentioned and discussed above, as well as their contrast to FDA-approved drugs (see sections 3.2 and 3.3.3).

### Chemical Multiverse Visualization

**Figure 9:**
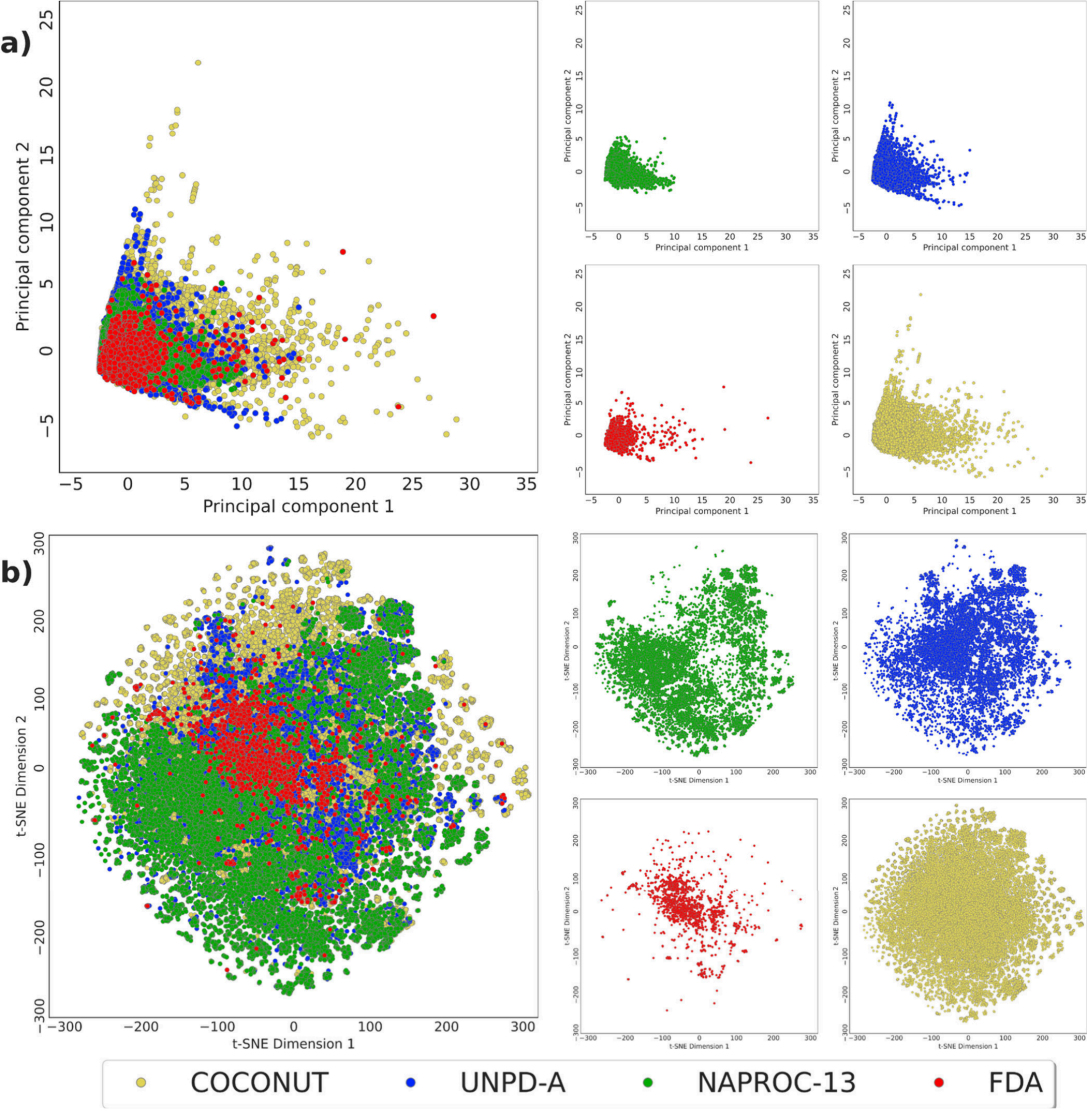
Chemical multiverse visualization of NPs in NAPROC-13 compared to those of UNPD-A, FDA-approved drugs, and COCONUT as the chemical space of NPs. (a) PCA of molecular descriptors of pharmacological interest (HBA, HBD, MW, TPSA, RB, LogP) and (b) t-SNE of structural fingerprint ECFP4.

Figure 9 shows a visual representation of the chemical multiverse of NAPROC-13, immersed in the chemical multiverse of the known NPs (using COCONUT as an approach to this set) and compared with the chemical multiverse of a set of NPs designed to be diverse, as is UNPD-A, and the FDA-approved drugs. According to physicochemical properties related to pharmacological interest, NAPROC-13 comprises a region of the chemical space shorter than UNPD-A, and both of them cover a limited region of NP chemical space. FDA-approved drugs overlap a considerable zone of NPs’ chemical space as has been demonstrated in previous analyses (see Figure 9A).^42^

According to their structural motifs, the coverage of the chemical space of NPs included in NAPROC-13 was approached using the molecular fingerprint ECFP4, broadly used in the representation of NPs in previous studies, and classically recognized as adaptable and functional for the correct representations of both small and synthetical molecules, as well as big molecules and of natural sources.^42,64^ The structural diversity of NPs present in NAPROC-13 determined by using this methodology, saving the character of UNPD-A of being a diversity-focused library as mentioned above, is highly remarkable (see Figure 9B).^2,42^

t-SNE reduction of components (perplexity = 30, learning rate = by default, number of iterations = 5000) performed well in the task of representing the chemical space covered by the present study, in the sense that small and synthetic molecules, such as FDA-approved drugs, were proximately clustered in the middle of the graphical representation, in contrast to natural products, that covered a broader region of the structural features. This trend appears to be reproduced in terms of druglike properties, where UNPD-A reflects a wider region of NP chemical space than NAPROC-13.

### Natural Product Likeness Score

**Figure 10:**
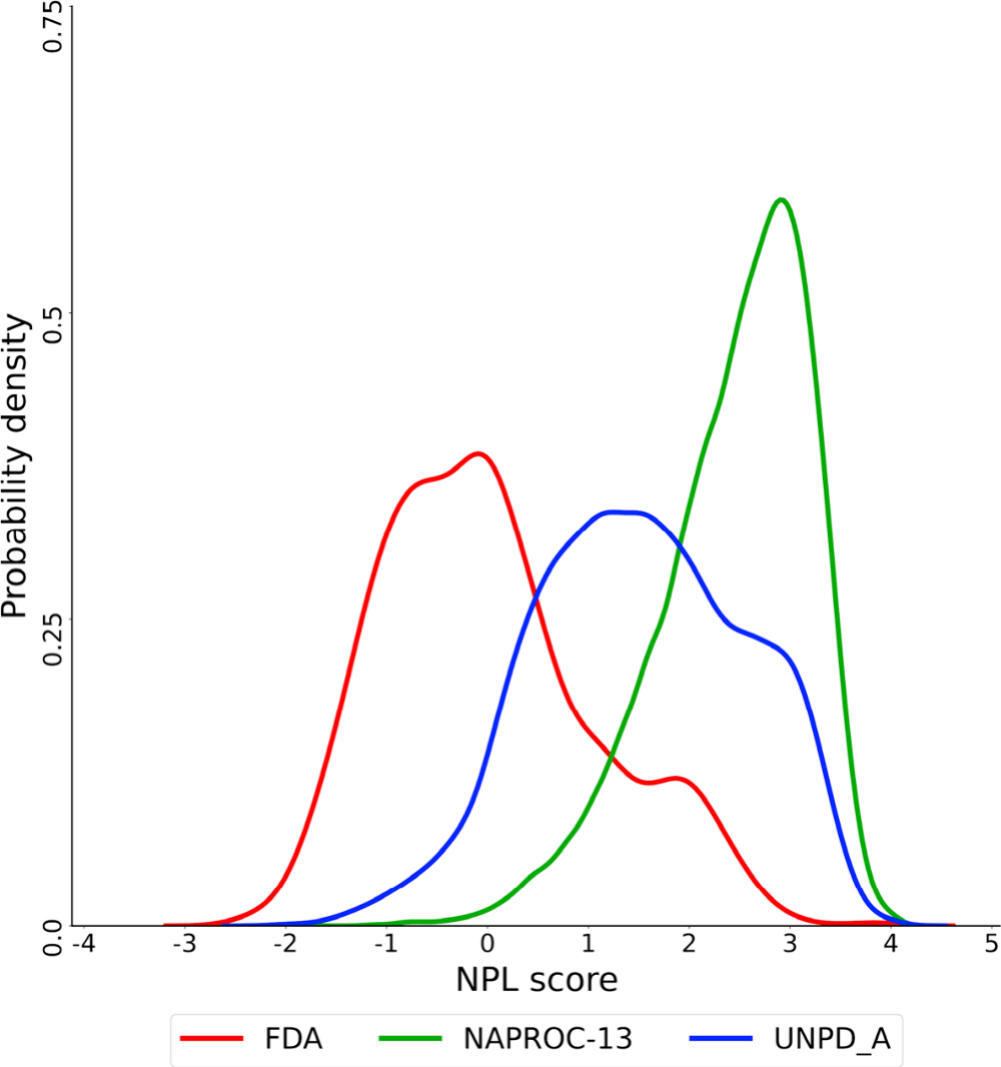
Distribution of the probability density of the Natural Product Likeness score of NPs in NAPROC-13, UNPD-A, and FDA-approved drugs.

Figure 10 shows the distribution of NPL scores of the compounds in different databases analyzed.^65^ The NPL score is an approximate measure to describe and summarize the characteristics of NPs that differentiate them from synthetic molecules and can be attached by different approaches and model methodologies. The approach used here consists of the most classical method and is based on a Bayesian statistical model based on fragmentation patterns of common apparition in NPs. Positive values of the NPL score mean that the chemical structure of a compound resembles an NP structure (given the data set used to train the metric). In contrast, negative values of the score mean that the chemical is more associated with fragmentation patterns of the synthetic organic compounds. In this study, profiling compound databases in terms of NPL scores aims to prioritize databases in terms of their coverage of the chemical space of NPs. Results in Figure 10 indicate that compounds in NAPROC-13 have large variability in terms of natural product likeness, but most of them, as expected, have a strong character of natural products: their NPL scores were highly shifted toward positive values (average: 2.44; median: 2.58; standard deviation: 0.8). Results also indicated that compounds NAPROC-13 have stronger features of NPs as compared to the NPs in UNPD-A (average: 1.51; median: 1.51; standard deviation: 1.1). As expected, both NP collections had higher values than compounds in the set of FDA-approved drugs (average: 0.02; median: −0.10; standard deviation: 1.1), which was used as a reference in the comparison. The distribution of NPL scores in Figure 10 agreed with the values calculated previously for other NPs and approved drugs.^39,66^

## Conclusions

NAPROC-13 is a freely accessible compound database containing over 24 000 NPs, with detailed information on their chemical structures and ^13^C NMR data. Notably, more than 4000 compounds in NAPROC-13 have yet to be included in large NP databases, such as COCONUT, highlighting its unique contribution to the field. Our analysis shows that the chemical structures of NPs in NAPROC-13 exhibit similar physicochemical and constitutional properties to those in a broad and diverse set of NPs. However, they also contain distinct molecular fragments, scaffolds, and ring systems of pharmaceutical relevance, distinguishing them from other databases.

The high structural complexity of NAPROC-13 NPs, compared to other screening libraries, including those of generally diverse NPs, underscores its potential for use in virtual screening for hit identification in drug discovery projects. While the chemical space of NAPROC-13, as described by drug-type properties, is somewhat limited to areas associated with approved drugs, its extensive distribution across the NP chemical space characterized by structural features makes it a valuable resource for identifying potential candidates in drug discovery efforts, especially for exploring under-represented areas of chemical diversity. This is further supported by our findings, where NAPROC-13 achieved higher NPL scores than those of other NP sets and approved drugs, demonstrating its significant potential in the field.

Moving forward, one of the key perspectives of this work is to enhance the NAPROC-13 web site by incorporating interactive features that display computed descriptors, graphics, scaffolds, and substructure searching tools. These updates will provide researchers with an invaluable resource for NP dereplication, drug design, and structure-based investigations. By continuously updating and expanding the database with new tools and data, we aim to further support NP research and facilitate advancements in spectroscopy, machine learning, and drug discovery.

## Experimental Section

### Data Sets and Standardization of Chemical Structures

At the time of writing (May 2024), NAPROC-13 had 24 722 compounds. While most entries in this web-based application originate from plant sources, it also includes some marine and microbiological NPs, reflecting recent revisions in their structures. It is noteworthy that the priority for entries in NAPROC-13 currently lies with substances of which a considerable number of thier structures have been reviewed.^21−23^ By geographical distribution, systematic introductions of NPs are limited to those from Panama and El Salvador. Most of the entries in NAPROC-13 come from various unspecified countries worldwide. For comparisons, we used the following as reference compound databases: the FDA set (update until January 4, 2023)^67,68^ with 2324 unique compounds, and the Universal Natural Product Database - Subset A (UNPD-A), which includes the 14 994 most diverse compounds from NPs reported in the UNPD, selected using the MaxMin algorithm.^2,69^ In addition, a set of reported solvents of analysis for NAPROC-13 compound analyses was created from the original and curated database as an approximation to the solubility of the chemical compounds in deuterated solvents. The COCONUT database was included as an approximation to the whole reported compounds of natural origin, to estimate the region of the chemical space of NPs that is covered by NAPROC-13.^8^

Compounds in NAPROC-13, UNPD-A, and FDA-approved drugs encoded as Simplified Molecular Input Line Entry System (SMILES)^70^ were standardized using the open-source cheminformatics toolkit RDKit, version 2023.09.4^71^ and MolVS.^72^ According to a standardized protocol,^73^ the functions Standardizer, LargestFragmentChoser, Uncharger, Reionizer, and TautomerCanonicalizer implemented in MolVS were used. Compounds with valence errors or any chemical element different from H, B, C, N, O, F, Si, P, S, Cl, Se, Br, and I were removed. Stereochemistry information was kept except for the computation of unique compounds, molecular scaffolds, and ring systems. Compounds with multiple components, if present, were split, and the largest component was retained. The remaining compounds were neutralized and reionized to generate the corresponding canonical tautomer.

### Molecular Descriptors

For each molecule, physicochemical properties of pharmaceutical interest, constitutional descriptors, and molecular fingerprints were calculated with Python language using RDKit toolkit version 2023.09.4^71^ and Molecular Operating Environment (MOE), version 2022.02.^74^

Descriptors computed with the RDKit toolkit were hydrogen bond acceptors (HBA), hydrogen bond donors (HBD), partition coefficient octanol/water (LogP), topological polar surface area (TPSA), molecular weight (MW), fraction of sp^3^ carbon atoms (CSP3), number of heavy atoms, number of ring systems, number of heteroatoms, number of rotatable bonds (RB), number of alicyclic rings formed by carbon atoms, number of alicyclic rings that include heteroatoms, number of aromatic rings formed by carbon atoms, number of aromatic rings that include heteroatoms, and the total number of aromatic rings. The number of acid atoms, aromatic atoms, basic atoms, nitrogen, oxygen, and halogen atoms, the fraction of rotatable bonds, and the number of chiral centers were computed using MOE.

Three types of molecular fingerprints with different designs were calculated: Molecular ACCes System (MACCS) keys (166-bits),^75^ extended connectivity fingerprint (ECFP)^76^ of 1024-bits with diameter 4 (ECFP4) and diameter 6 (ECFP6), and MinHashed atom pair fingerprint up to a diameter of four bonds (MAP4).^77,78^

### Structural Content and Diversity

The structural diversity of NPs reported in NAPROC-13 was analyzed by comparison with FDA-approved drugs and the most diverse set of NPs (UNPD-A), in terms of their distribution of similarity values, computed with the Tanimoto coefficient using four molecular fingerprints: MACCS Keys (166 bits), ECFP4, ECFP6, and MAP4. For NPs in NAPROC-13 and UNPD-A, five random samples of 1000 compounds each were extracted to reduce the computational cost. It has been demonstrated that multiple sampling of 1000 compounds is a valid approach to quantify the entire database pairwise fingerprint-based diversity.^79^

Among the multiple methods to perform the molecular scaffold analysis of a set of compounds,^80^ we used the definition proposed by Bemis and Murcko, which consists of removing all side chains in molecules and preserving the ring systems and their corresponding linkers.^26^ Along with this calculation, scaffold diversity was estimated by computing the scaled Shannon’s entropy (SSE) of molecular distribution along the set of presented scaffolds, taking into account the distribution along the first 15 scaffolds.^81^ The Cyclic System Retrieval (CSR) curve was generated as a visual guide to compare the relative scaffold diversity of the databases.

Also, we implemented ring systems to determine the presence of “Magic Rings” in NAPROC-13 and the reference databases. A ring system consists of a cyclic system without any bridge connecting to another cyclic ring, preserving exocyclic double bonds.^82^ This approach has proven to be highly efficient for the analysis of chemical structures.^83^ Ring systems were computed using the implementation of RingSystemFinder in the library useful_rdkit_utils.^84^ This algorithm identifies and protects exocyclic double bonds connected to rings, cleaves single bonds with the FragmentOnBonds RDKit function, and returns the cyclic fragments after the addition of hydrogen atoms to cleavage sites.

The REtrosynthetic Combinatorial Analysis Procedure (RECAP) algorithm,^27^ implemented in the RDKit package, was used to generate the molecular fragments in the studied databases. RECAP algorithm fragments molecular structures around bonds formed by common chemical reactions. Recognized cleavable bonds for the RECAP algorithm are amide, ester, amine, urea, ether, olefin, quaternary nitrogen, aromatic carbon, and sulfonamide. Those rules are applied for acyclic bonds, while rings are maintained intact. In the present work, we compared the content of fragments of NPs lighter than 1350 g/mol in NAPROC-13 (21 226 compounds) with those generated from NPs lighter than 1000 g/mol in UNPD-A (14 733 compounds) and FDA-approved drugs lighter than 2000 g/mol (2317 compounds). MW filtering was applied to reduce the computational cost, and the benchmark was designated according to the time of computation related to the molecular complexity. The newly generated fragment library is freely available at https://github.com/DIFACQUIM/naproc13_characterization. Finally, molecular descriptors related to the rule of three (RO3) were computed using the Datamol Python library, version 0.12.3.^60,85^

### Chemical Multiverse Visualization

The chemical multiverses of the three databases, NAPROC-13, UNPD-A, and FDA-approved drugs, were compared against the chemical space of NPs, using COCONUT as an approach to these sets. A chemical multiverse is a group of alternative or “parallel” chemical spaces of a set of compounds, each defined by a distinct set of molecular descriptors.^29^ Each chemical space is an M-dimensional Cartesian space, and each dimension represents the descriptors or features encoding a molecule. The length of the descriptor sets defines the number of dimensions of each chemical space. Dimensionality reduction for chemical space visualization was achieved using t-distributed stochastic neighbor embedding (t-SNE) according to the bits-based fingerprints previously computed (vide supra). t-SNE is a nonlinear method that uses t-distribution instead of the linear method used by the principal component analysis (PCA). This approach allows t-SNE to display a wider distribution of points along the graph.^86^ A PCA using the most significant physicochemical and constitutional descriptors was also computed. PCA and t-SNE variable reductions were done using the library Scikit-Learn 1.4.1.^87^

### Natural Product Likeness Score

The Natural Product Likeness (NPL) score^65^ is a machine-learning-based scoring that quantifies how similar a compound is to the structural chemical space covered by those NPs. This approach efficiently distinguishes NPs from synthetic molecules. It has been broadly used to classify compounds of natural origin^2,42^ and food components^39^ and to validate the NPL of machine learning-based generative models.^88^ The index consists of a range between −5 (for compounds of probable synthetic origin) and 5 (for compounds similar to an NP). In the present work, we employed the NPL score for the compounds in NAPROC-13, as well as UNPD-A and FDA-approved drugs.

## Data Availability

Data sets and codes are freely available at https://github.com/DIFACQUIM/naproc13_characterization.

## Supporting Information Available

Details of the comprehensive chemoinformatic characterization as well as machine learning approach analysis of NAPROC-13 versus FDA-approved drugs and UNPD-A, and data visualization (PDF)
