# Supplementary material for: Chemoinformatic Characterization of NAPROC-13: A Database for Natural Product 13C NMR Dereplication
Source: J Nat Prod. 2024 Sep 13;87(9):2216–29. doi: 10.1021/acs.jnatprod.4c00530 (PMC11443490; doi:10.1021/acs.jnatprod.4c00530)
Supplement: Supplementary file 1 — np4c00530_si_001.pdf [file np4c00530_si_001.pdf]

**SUPPORTING INFORMATION**

# Chemoinformatic characterization of NAPROC-13: A database for natural product $^{13}C$ NMR dereplication

Juan F. Avellaneda-Tamayo[1], Naicolette A. Agudo-Muñoz[2,4], Javier E. Sánchez-Galán[3,4], José Luis López-Pérez[5,6,*], José L. Medina-Franco[1*]

[1]*DIFACQUIM Research Group, Department of Pharmacy, School of Chemistry, Universidad Nacional Autónoma de México, Avenida Universidad 3000, Mexico City 04510, Mexico*

[2]*Science and Technology Faculty, Universidad Tecnológica de Panamá, Campus Metropolitano Víctor Levi Sasso, Avenida Universidad Tecnológica, Vía Puente Centenario, Panama City, Panama*

[3]*Facultad de Ingeniería de Sistemas Computacionales, Universidad Tecnológica de Panamá, Campus Metropolitano Víctor Levi Sasso, Avenida Universidad Tecnológica, Vía Puente Centenario, Panama City, Panama*

[4]*Grupo de Investigación en Biotecnología, Bioinformática y Biología de Sistemas (GIBBS), Universidad Tecnológica de Panama*

[5]*Departamento de Ciencias Farmacéuticas, Área de Química Farmacéutica, Facultad de Farmacia, CIETUS, IBSAL, Campus Miguel de Unamuno, University of Salamanca, 37007, Salamanca, Spain*

[6]*Departamento de Farmacología, Facultad de Medicina, CIPFAR, Universidad de Panamá, Panama City, Panama*

[*]Correspondence authors: medinajl@unam.mx, Tel.: +52-55-5622-3899 (JLMF); lopez@usal.es (JLLP)

# CONTENTS


| | | Page |
|---|---|---|
| **Figure S1** | Unique and overlapping content among natural products in NAPROC-13 (green), UNPD-A (blue), and FDA-approved drugs (red). Structural content was analyzed in terms of (a) entire compounds and (b) molecular scaffolds. | S3 |
| **Table S1** | Descriptive statistics of physicochemical and constitutional descriptors computed for NAPROC-13 compounds, FDA-approved drugs, and natural products in UNPD-A. | S4 |
| **Figure S2** | Distribution of physicochemical properties and constitutional descriptors of interest among NAPROC-13 compounds, FDA-approved drugs, and natural products in UNPD-A. | S8 |
| **Table S2** | Descriptive statistics of physicochemical and constitutional descriptors computed for NAPROC-13 among their solubility categories. | S9 |
| **Figure S3** | Distribution of physicochemical properties and constitutional descriptors of interest computed for NAPROC-13 among their solubility categories. | S16 |
| **Table S3** | Descriptive statistics of pairwise similarity distribution computed for NPs in NAPROC-13 and UNPDA, and FDA-approved drugs. | S18 |
| **Table S4** | Descriptive statistics of natural product-likeness scores computed for food components (FooDB), natural products (UNPD-A), FDA-approved drugs, and commercially available compounds from FooDB. | S19 |
| **Figure S4** | Distribution of the “magic-ring systems” in NAPROC-13, FDA-approved drugs, and UNPD-A, among the classes of biological targets predicted to be most probable to have biological activity. The most common category was the “not known” target, with 728 ring systems in NAPROC-13, 33 in FDA-approved drugs, and 570 in UNPD-A. | S20 |
| **Figure S5** | Most frequent potentially bioactive ring systems in FDA-approved drugs (“magic rings”). | S21 |
| **Figure S6** | Most frequent potentially bioactive ring systems in UNPD-A (“magic rings”). | S21 |

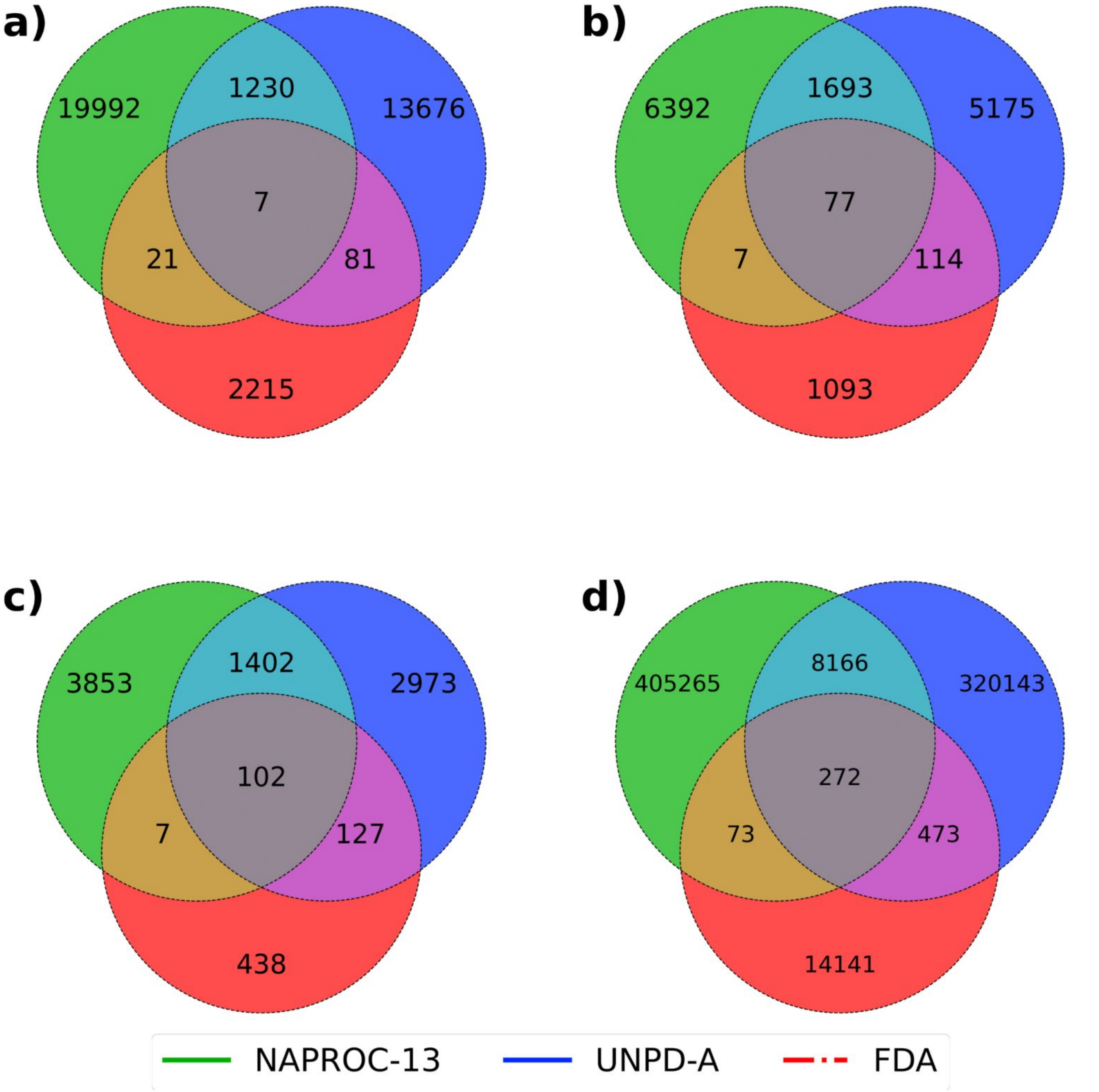


**Figure S1.** Unique and overlapping content among NPs in NAPROC-13 (green), UNPD-A (blue), and FDA-approved drugs (red). Structural content was analyzed in terms of (a) entire compounds, (b) molecular scaffolds, (c) ring systems, and (d) molecular fragments.

**Table S1.** Descriptive statistics of physicochemical and constitutional descriptors computed for NAPROC-13 compounds, FDA-approved drugs, and NPs in UNPD-A.

| descriptor | data set | number of compounds | mean | std | min | Q1 | median | Q3 | max |
|---|---|---|---|---|---|---|---|---|---|
| Number of acidic atoms | NAPROC-13 | 21250 | 0.00 | 0.06 | 0.00 | 0.00 | 0.00 | 0.00 | 5.00 |
| | FDA | 2324 | 0.12 | 0.71 | 0.00 | 0.00 | 0.00 | 0.00 | 12.00 |
| | UNPD-A | 14994 | 0.00 | 0.00 | 0.00 | 0.00 | 0.00 | 0.00 | 0.00 |
| Number of aromatic rings | NAPROC-13 | 21250 | 0.75 | 1.09 | 0.00 | 0.00 | 0.00 | 1.00 | 8.00 |
| | FDA | 2324 | 1.54 | 1.31 | 0.00 | 0.00 | 1.00 | 2.00 | 10.00 |
| | UNPD-A | 14994 | 1.28 | 1.49 | 0.00 | 0.00 | 1.00 | 2.00 | 15.00 |
| Number of aromatic atoms | NAPROC-13 | 21250 | 3.56 | 5.17 | 0.00 | 0.00 | 0.00 | 6.00 | 36.00 |
| | FDA | 2324 | 7.63 | 6.74 | 0.00 | 0.00 | 6.00 | 12.00 | 60.00 |
| | UNPD-A | 14994 | 6.19 | 7.43 | 0.00 | 0.00 | 6.00 | 12.00 | 84.00 |
| Number of basic atoms | NAPROC-13 | 21250 | 0.00 | 0.07 | 0.00 | 0.00 | 0.00 | 0.00 | 5.00 |
| | FDA | 2324 | 0.05 | 0.27 | 0.00 | 0.00 | 0.00 | 0.00 | 4.00 |
| | UNPD-A | 14994 | 0.00 | 0.00 | 0.00 | 0.00 | 0.00 | 0.00 | 0.00 |
| Number of bromine atoms | NAPROC-13 | 21250 | 0.01 | 0.15 | 0.00 | 0.00 | 0.00 | 0.00 | 3.00 |
| | FDA | 2324 | 0.02 | 0.17 | 0.00 | 0.00 | 0.00 | 0.00 | 4.00 |
| | UNPD-A | 14994 | 0.05 | 0.40 | 0.00 | 0.00 | 0.00 | 0.00 | 7.00 |
| Number of alicyclic rings of carbon | NAPROC-13 | 21250 | 2.27 | 1.71 | 0.00 | 1.00 | 2.00 | 4.00 | 10.00 |
| | FDA | 2324 | 0.51 | 1.13 | 0.00 | 0.00 | 0.00 | 0.00 | 6.00 |

| | | | | | | | | | |
|---|---|---|---|---|---|---|---|---|---|
| | UNPD-A | 14994 | 0.96 | 1.48 | 0.00 | 0.00 | 0.00 | 2.00 | 10.00 |
| Number of aromatic rings of carbon | NAPROC-13 | 21250 | 0.51 | 0.86 | 0.00 | 0.00 | 0.00 | 1.00 | 6.00 |
| | FDA | 2324 | 1.03 | 1.00 | 0.00 | 0.00 | 1.00 | 2.00 | 10.00 |
| | UNPD-A | 14994 | 0.96 | 1.27 | 0.00 | 0.00 | 0.00 | 2.00 | 14.00 |
| Number of chiral centers | NAPROC-13 | 21250 | 6.59 | 5.07 | 0.00 | 3.00 | 6.00 | 9.00 | 40.00 |
| | FDA | 2324 | 2.31 | 3.83 | 0.00 | 0.00 | 1.00 | 3.00 | 41.00 |
| | UNPD-A | 14994 | 3.81 | 5.13 | 0.00 | 0.00 | 2.00 | 6.00 | 43.00 |
| Number of chlorine atoms | NAPROC-13 | 21250 | 0.02 | 0.17 | 0.00 | 0.00 | 0.00 | 0.00 | 5.00 |
| | FDA | 2324 | 0.20 | 0.59 | 0.00 | 0.00 | 0.00 | 0.00 | 6.00 |
| | UNPD-A | 14994 | 0.04 | 0.34 | 0.00 | 0.00 | 0.00 | 0.00 | 10.00 |
| CSP3 | NAPROC-13 | 21250 | 0.67 | 0.24 | 0.00 | 0.54 | 0.72 | 0.85 | 1.00 |
| | FDA | 2324 | 0.45 | 0.27 | 0.00 | 0.26 | 0.43 | 0.63 | 1.00 |
| | UNPD-A | 14994 | 0.52 | 0.31 | 0.00 | 0.25 | 0.52 | 0.80 | 1.00 |
| Number of fluorine atoms | NAPROC-13 | 21250 | 0.00 | 0.09 | 0.00 | 0.00 | 0.00 | 0.00 | 6.00 |
| | FDA | 2324 | 0.28 | 0.96 | 0.00 | 0.00 | 0.00 | 0.00 | 14.00 |
| | UNPD-A | 14994 | 0.00 | 0.05 | 0.00 | 0.00 | 0.00 | 0.00 | 3.00 |
| Fraction of rotatable bonds | NAPROC-13 | 21250 | 0.13 | 0.10 | 0.00 | 0.06 | 0.12 | 0.18 | 0.87 |
| | FDA | 2324 | 0.23 | 0.15 | 0.00 | 0.12 | 0.20 | 0.30 | 0.95 |
| | UNPD-A | 14994 | 0.19 | 0.20 | 0.00 | 0.05 | 0.13 | 0.24 | 0.97 |
| Number of halogen atoms | NAPROC-13 | 21250 | 0.04 | 0.27 | 0.00 | 0.00 | 0.00 | 0.00 | 6.00 |
| | FDA | 2324 | 0.55 | 1.20 | 0.00 | 0.00 | 0.00 | 1.00 | 14.00 |

| | | | | | | | | | |
|---|---|---|---|---|---|---|---|---|---|
| | UNPD-A | 14994 | 0.10 | 0.54 | 0.00 | 0.00 | 0.00 | 0.00 | 10.00 |
| HBA | NAPROC-13 | 21250 | 6.07 | 4.17 | 0.00 | 3.00 | 5.00 | 8.00 | 36.00 |
| | FDA | 2324 | 5.29 | 4.61 | 0.00 | 3.00 | 4.00 | 6.00 | 59.00 |
| | UNPD-A | 14994 | 5.58 | 4.95 | 0.00 | 2.00 | 4.00 | 7.00 | 53.00 |
| HBD | NAPROC-13 | 21250 | 2.27 | 2.44 | 0.00 | 1.00 | 2.00 | 3.00 | 20.00 |
| | FDA | 2324 | 2.45 | 3.70 | 0.00 | 1.00 | 2.00 | 3.00 | 57.00 |
| | UNPD-A | 14994 | 2.51 | 3.17 | 0.00 | 0.00 | 2.00 | 3.00 | 36.00 |
| Number of heavy atoms | NAPROC-13 | 21250 | 30.77 | 11.49 | 1.00 | 23.00 | 29.00 | 36.00 | 112.00 |
| | FDA | 2324 | 26.68 | 18.86 | 1.00 | 17.00 | 23.00 | 31.00 | 291.00 |
| | UNPD-A | 14994 | 26.38 | 13.90 | 1.00 | 18.00 | 24.00 | 32.00 | 135.00 |
| Number of heteroatoms | NAPROC-13 | 21250 | 6.25 | 4.19 | 0.00 | 3.00 | 5.00 | 8.00 | 36.00 |
| | FDA | 2324 | 7.50 | 6.97 | 0.00 | 4.00 | 6.00 | 9.00 | 106.00 |
| | UNPD-A | 14994 | 6.02 | 5.08 | 0.00 | 3.00 | 5.00 | 7.00 | 53.00 |
| Number of alicyclic rings with heteroatoms | NAPROC-13 | 21250 | 0.90 | 1.15 | 0.00 | 0.00 | 1.00 | 1.00 | 13.00 |
| | FDA | 2324 | 0.73 | 1.21 | 0.00 | 0.00 | 0.00 | 1.00 | 30.00 |
| | UNPD-A | 14994 | 0.86 | 1.26 | 0.00 | 0.00 | 0.00 | 1.00 | 21.00 |
| Number of aromatic rings with heteroatoms | NAPROC-13 | 21250 | 0.24 | 0.46 | 0.00 | 0.00 | 0.00 | 0.00 | 4.00 |
| | FDA | 2324 | 0.51 | 0.77 | 0.00 | 0.00 | 0.00 | 1.00 | 6.00 |
| | UNPD-A | 14994 | 0.32 | 0.60 | 0.00 | 0.00 | 0.00 | 1.00 | 10.00 |
| Number of | NAPROC-13 | 21250 | 0.00 | 0.04 | 0.00 | 0.00 | 0.00 | 0.00 | 2.00 |

| | | | | | | | | | |
|---|---|---|---|---|---|---|---|---|---|
| iodine atoms | FDA | 2324 | 0.05 | 0.40 | 0.00 | 0.00 | 0.00 | 0.00 | 6.00 |
| | UNPD-A | 14994 | 0.00 | 0.07 | 0.00 | 0.00 | 0.00 | 0.00 | 3.00 |
| CLogP | NAPROC-13 | 21250 | 3.54 | 2.41 | -8.04 | 2.15 | 3.48 | 4.91 | 15.73 |
| | FDA | 2324 | 2.27 | 2.87 | -23.83 | 0.70 | 2.55 | 3.94 | 18.55 |
| | UNPD-A | 14994 | 2.94 | 3.02 | -18.53 | 1.46 | 2.87 | 4.32 | 24.43 |
| MW | NAPROC-13 | 21250 | 430.38 | 163.63 | 16.04 | 317.83 | 404.46 | 502.52 | 1597.71 |
| | FDA | 2324 | 387.38 | 271.96 | 12.01 | 247.95 | 337.37 | 447.14 | 4113.64 |
| | UNPD-A | 14994 | 371.94 | 196.43 | 16.04 | 246.31 | 330.29 | 445.59 | 1887.28 |
| Number of nitrogen atoms | NAPROC-13 | 21250 | 0.06 | 0.32 | 0.00 | 0.00 | 0.00 | 0.00 | 5.00 |
| | FDA | 2324 | 2.54 | 3.27 | 0.00 | 1.00 | 2.00 | 3.00 | 51.00 |
| | UNPD-A | 14994 | 0.48 | 1.21 | 0.00 | 0.00 | 0.00 | 0.00 | 18.00 |
| Number of oxygen atoms | NAPROC-13 | 21250 | 6.14 | 4.18 | 0.00 | 3.00 | 5.00 | 8.00 | 36.00 |
| | FDA | 2324 | 4.04 | 4.45 | 0.00 | 2.00 | 3.00 | 5.00 | 59.00 |
| | UNPD-A | 14994 | 5.38 | 5.05 | 0.00 | 2.00 | 4.00 | 7.00 | 53.00 |
| Number of phosphorus atoms | NAPROC-13 | 21250 | 0.00 | 0.02 | 0.00 | 0.00 | 0.00 | 0.00 | 1.00 |
| | FDA | 2324 | 0.04 | 0.26 | 0.00 | 0.00 | 0.00 | 0.00 | 4.00 |
| | UNPD-A | 14994 | 0.00 | 0.03 | 0.00 | 0.00 | 0.00 | 0.00 | 1.00 |
| Number of ring systems | NAPROC-13 | 21250 | 3.92 | 1.72 | 0.00 | 3.00 | 4.00 | 5.00 | 17.00 |
| | FDA | 2324 | 2.78 | 1.98 | 0.00 | 1.00 | 3.00 | 4.00 | 30.00 |
| | UNPD-A | 14994 | 3.09 | 2.19 | 0.00 | 2.00 | 3.00 | 4.00 | 21.00 |

| | | | | | | | | | |
|---|---|---|---|---|---|---|---|---|---|
| Number of rotable bonds | NAPROC-13 | 21250 | 3.96 | 3.51 | 0.00 | 1.00 | 3.00 | 6.00 | 51.00 |
| | FDA | 2324 | 5.98 | 7.63 | 0.00 | 2.00 | 4.00 | 7.00 | 149.00 |
| | UNPD-A | 14994 | 4.74 | 6.02 | 0.00 | 1.00 | 3.00 | 6.00 | 59.00 |
| TPSA | NAPROC-13 | 21250 | 96.13 | 64.07 | 0.00 | 55.76 | 80.92 | 118.22 | 557.71 |
| | FDA | 2324 | 95.71 | 106.28 | 0.00 | 43.37 | 74.60 | 110.77 | 1690.64 |
| | UNPD-A | 14994 | 90.78 | 82.74 | 0.00 | 40.46 | 69.67 | 112.05 | 877.36 |

[a] std: standard deviation.

[b] min: minimum value.

[c] Q1: value under which 25% of data points are found in increasing order.

[d] Q3: value under which 75% of data points are found in increasing order.

[e] max: maximum value.

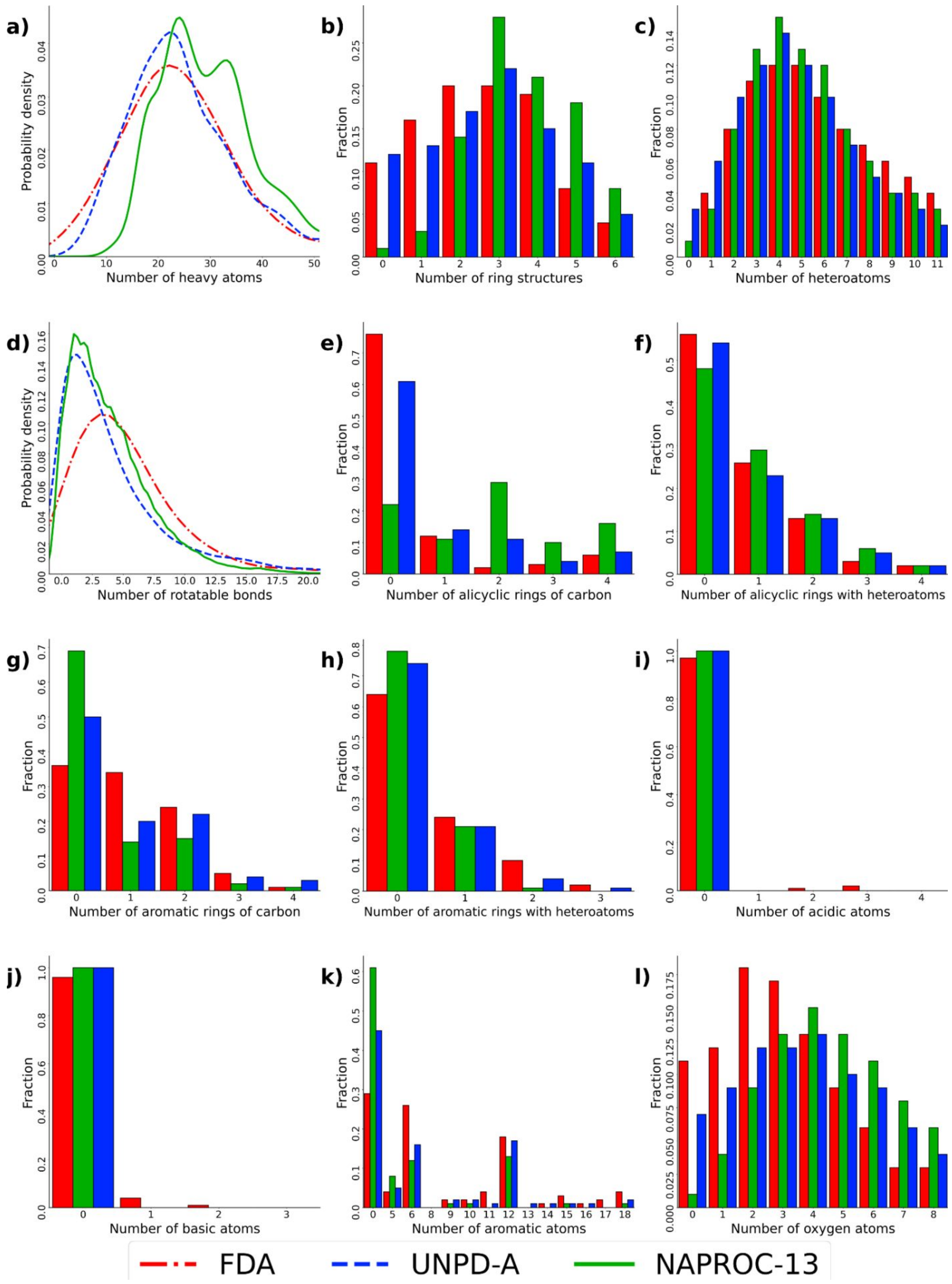


**Figure S2.** Distribution of physicochemical properties and constitutional descriptors of interest among NAPROC-13 compounds, FDA-approved drugs, and NPs in UNPD-A. Dotted lines are used for ease of visualization.

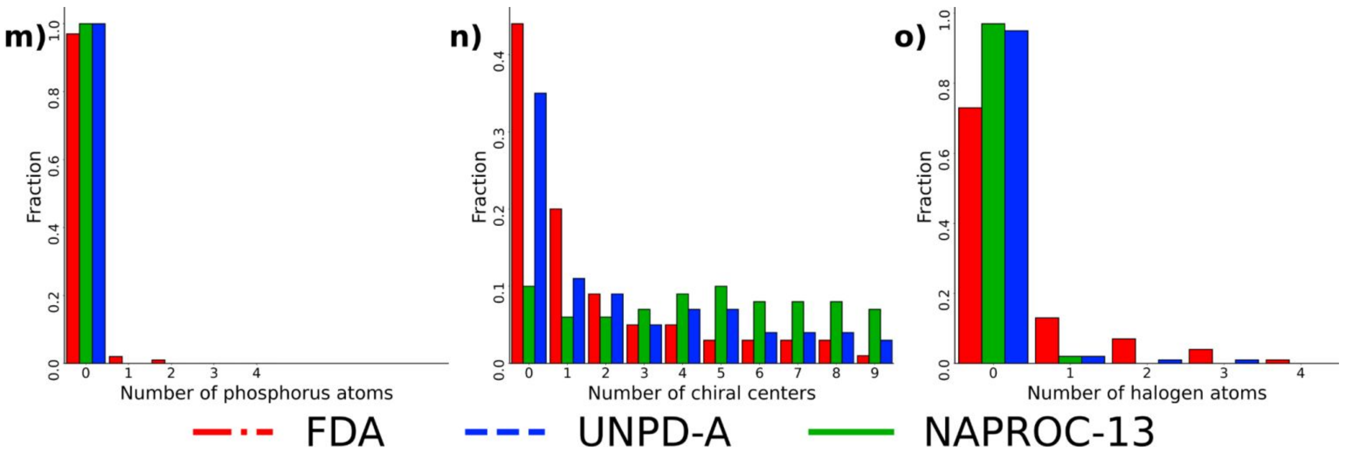


**Figure S2 (cont).** Distribution of physicochemical properties and constitutional descriptors of interest among NAPROC-13 compounds, FDA-approved drugs, and NPs in UNPD-A. Dotted lines are used for ease of visualization.

**Table S2.** Descriptive statistics of physicochemical and constitutional descriptors computed for NAPROC-13 among their solubility categories.

| descriptor | data set | number of compounds | mean | std[a] | min[b] | Q1[c] | median | Q3[d] | max[e] |
|---|---|---|---|---|---|---|---|---|---|
| Number of acidic atoms | Acetone | 822 | 0.00 | 0.00 | 0.00 | 0.00 | 0.00 | 0.00 | 0.00 |
| | Benzene | 309 | 0.00 | 0.06 | 0.00 | 0.00 | 0.00 | 0.00 | 1.00 |
| | Chloroform | 13481 | 0.00 | 0.04 | 0.00 | 0.00 | 0.00 | 0.00 | 2.00 |
| | DMSO | 1415 | 0.00 | 0.14 | 0.00 | 0.00 | 0.00 | 0.00 | 5.00 |
| | Methanol | 2060 | 0.00 | 0.00 | 0.00 | 0.00 | 0.00 | 0.00 | 0.00 |
| | Pyridine | 2496 | 0.00 | 0.00 | 0.00 | 0.00 | 0.00 | 0.00 | 0.00 |
| Number of aromatic rings | Acetone | 822 | 1.88 | 1.34 | 0.00 | 1.00 | 2.00 | 3.00 | 6.00 |
| | Benzene | 309 | 0.26 | 0.57 | 0.00 | 0.00 | 0.00 | 0.00 | 4.00 |

| | | | | | | | | |
|---|---|---|---|---|---|---|---|---|
| | Chloroform | 13481 | 0.61 | 0.94 | 0.00 | 0.00 | 0.00 | 1.00 | 6.00 |
| | DMSO | 1415 | 2.04 | 1.33 | 0.00 | 1.00 | 2.00 | 3.00 | 8.00 |
| | Methanol | 2060 | 0.94 | 1.16 | 0.00 | 0.00 | 1.00 | 2.00 | 6.00 |
| | Pyridine | 2496 | 0.24 | 0.65 | 0.00 | 0.00 | 0.00 | 0.00 | 5.00 |
| Number of aromatic atoms | Acetone | 822 | 8.98 | 6.47 | 0.00 | 5.00 | 12.00 | 12.00 | 36.00 |
| | Benzene | 309 | 1.25 | 2.73 | 0.00 | 0.00 | 0.00 | 0.00 | 15.00 |
| | Chloroform | 13481 | 2.99 | 4.58 | 0.00 | 0.00 | 0.00 | 6.00 | 28.00 |
| | DMSO | 1415 | 8.61 | 6.01 | 0.00 | 0.00 | 12.00 | 12.00 | 36.00 |
| | Methanol | 2060 | 4.53 | 5.85 | 0.00 | 0.00 | 0.00 | 6.00 | 36.00 |
| | Pyridine | 2496 | 1.11 | 3.03 | 0.00 | 0.00 | 0.00 | 0.00 | 24.00 |
| Number of basic atoms | Acetone | 822 | 0.00 | 0.00 | 0.00 | 0.00 | 0.00 | 0.00 | 0.00 |
| | Benzene | 309 | 0.00 | 0.06 | 0.00 | 0.00 | 0.00 | 0.00 | 1.00 |
| | Chloroform | 13481 | 0.00 | 0.05 | 0.00 | 0.00 | 0.00 | 0.00 | 2.00 |
| | DMSO | 1415 | 0.00 | 0.14 | 0.00 | 0.00 | 0.00 | 0.00 | 5.00 |
| | Methanol | 2060 | 0.01 | 0.11 | 0.00 | 0.00 | 0.00 | 0.00 | 2.00 |
| | Pyridine | 2496 | 0.00 | 0.00 | 0.00 | 0.00 | 0.00 | 0.00 | 0.00 |
| Number of bromine atoms | Acetone | 822 | 0.01 | 0.11 | 0.00 | 0.00 | 0.00 | 0.00 | 2.00 |
| | Benzene | 309 | 0.03 | 0.22 | 0.00 | 0.00 | 0.00 | 0.00 | 2.00 |
| | Chloroform | 13481 | 0.02 | 0.18 | 0.00 | 0.00 | 0.00 | 0.00 | 3.00 |

| | | | | | | | | | |
|---|---|---|---|---|---|---|---|---|---|
| | DMSO | 1415 | 0.00 | 0.07 | 0.00 | 0.00 | 0.00 | 0.00 | 2.00 |
| | Methanol | 2060 | 0.01 | 0.11 | 0.00 | 0.00 | 0.00 | 0.00 | 2.00 |
| | Pyridine | 2496 | 0.00 | 0.02 | 0.00 | 0.00 | 0.00 | 0.00 | 1.00 |
| Number of alicyclic rings of carbon | Acetone | 822 | 0.95 | 1.40 | 0.00 | 0.00 | 0.00 | 2.00 | 7.00 |
| | Benzene | 309 | 1.96 | 1.00 | 0.00 | 1.00 | 2.00 | 3.00 | 5.00 |
| | Chloroform | 13481 | 2.31 | 1.59 | 0.00 | 1.00 | 2.00 | 4.00 | 9.00 |
| | DMSO | 1415 | 0.92 | 1.50 | 0.00 | 0.00 | 0.00 | 2.00 | 7.00 |
| | Methanol | 2060 | 1.93 | 1.67 | 0.00 | 0.00 | 2.00 | 3.00 | 7.00 |
| | Pyridine | 2496 | 3.67 | 1.49 | 0.00 | 3.00 | 4.00 | 5.00 | 10.00 |
| Number of aromatic rings of carbon | Acetone | 822 | 1.49 | 1.11 | 0.00 | 0.00 | 2.00 | 2.00 | 6.00 |
| | Benzene | 309 | 0.11 | 0.41 | 0.00 | 0.00 | 0.00 | 0.00 | 3.00 |
| | Chloroform | 13481 | 0.40 | 0.75 | 0.00 | 0.00 | 0.00 | 1.00 | 6.00 |
| | DMSO | 1415 | 1.43 | 1.04 | 0.00 | 0.00 | 2.00 | 2.00 | 6.00 |
| | Methanol | 2060 | 0.67 | 0.97 | 0.00 | 0.00 | 0.00 | 1.00 | 6.00 |
| | Pyridine | 2496 | 0.14 | 0.48 | 0.00 | 0.00 | 0.00 | 0.00 | 4.00 |
| Number of chiral centers | Acetone | 822 | 3.13 | 3.51 | 0.00 | 0.00 | 2.00 | 5.00 | 23.00 |
| | Benzene | 309 | 4.38 | 2.42 | 0.00 | 3.00 | 4.00 | 6.00 | 14.00 |
| | Chloroform | 13481 | 5.85 | 3.61 | 0.00 | 3.00 | 6.00 | 8.00 | 33.00 |
| | DMSO | 1415 | 4.43 | 4.89 | 0.00 | 0.00 | 4.00 | 8.00 | 30.00 |

| | | | | | | | | | |
|---|---|---|---|---|---|---|---|---|---|
| | Methanol | 2060 | 7.76 | 5.77 | 0.00 | 4.00 | 7.00 | 10.00 | 39.00 |
| | Pyridine | 2496 | 12.48 | 6.99 | 0.00 | 8.00 | 11.00 | 16.00 | 40.00 |
| Number of chlorine atoms | Acetone | 822 | 0.01 | 0.08 | 0.00 | 0.00 | 0.00 | 0.00 | 1.00 |
| | Benzene | 309 | 0.01 | 0.11 | 0.00 | 0.00 | 0.00 | 0.00 | 1.00 |
| | Chloroform | 13481 | 0.03 | 0.20 | 0.00 | 0.00 | 0.00 | 0.00 | 5.00 |
| | DMSO | 1415 | 0.01 | 0.12 | 0.00 | 0.00 | 0.00 | 0.00 | 2.00 |
| | Methanol | 2060 | 0.01 | 0.11 | 0.00 | 0.00 | 0.00 | 0.00 | 4.00 |
| | Pyridine | 2496 | 0.00 | 0.07 | 0.00 | 0.00 | 0.00 | 0.00 | 1.00 |
| CSP3 | Acetone | 822 | 0.42 | 0.25 | 0.00 | 0.24 | 0.35 | 0.62 | 1.00 |
| | Benzene | 309 | 0.73 | 0.15 | 0.24 | 0.65 | 0.73 | 0.85 | 1.00 |
| | Chloroform | 13481 | 0.68 | 0.21 | 0.00 | 0.59 | 0.72 | 0.84 | 1.00 |
| | DMSO | 1415 | 0.41 | 0.28 | 0.00 | 0.17 | 0.33 | 0.65 | 1.00 |
| | Methanol | 2060 | 0.66 | 0.23 | 0.00 | 0.50 | 0.70 | 0.85 | 1.00 |
| | Pyridine | 2496 | 0.83 | 0.16 | 0.00 | 0.79 | 0.87 | 0.93 | 1.00 |
| Number of fluorine atoms | Acetone | 822 | 0.00 | 0.05 | 0.00 | 0.00 | 0.00 | 0.00 | 1.00 |
| | Benzene | 309 | 0.00 | 0.06 | 0.00 | 0.00 | 0.00 | 0.00 | 1.00 |
| | Chloroform | 13481 | 0.00 | 0.10 | 0.00 | 0.00 | 0.00 | 0.00 | 6.00 |
| | DMSO | 1415 | 0.00 | 0.00 | 0.00 | 0.00 | 0.00 | 0.00 | 0.00 |
| | Methanol | 2060 | 0.00 | 0.00 | 0.00 | 0.00 | 0.00 | 0.00 | 0.00 |

| | | | | | | | | | |
|---|---|---|---|---|---|---|---|---|---|
| | Pyridine | 2496 | 0.00 | 0.00 | 0.00 | 0.00 | 0.00 | 0.00 | 0.00 |
| Fraction of rotatable bonds | Acetone | 822 | 0.12 | 0.08 | 0.00 | 0.06 | 0.11 | 0.17 | 0.59 |
| | Benzene | 309 | 0.11 | 0.10 | 0.00 | 0.04 | 0.08 | 0.17 | 0.53 |
| | Chloroform | 13481 | 0.14 | 0.11 | 0.00 | 0.05 | 0.12 | 0.19 | 0.87 |
| | DMSO | 1415 | 0.11 | 0.07 | 0.00 | 0.06 | 0.11 | 0.16 | 0.50 |
| | Methanol | 2060 | 0.15 | 0.10 | 0.00 | 0.08 | 0.14 | 0.20 | 0.75 |
| | Pyridine | 2496 | 0.11 | 0.07 | 0.00 | 0.05 | 0.11 | 0.16 | 0.83 |
| Number of halogen atoms | Acetone | 822 | 0.02 | 0.14 | 0.00 | 0.00 | 0.00 | 0.00 | 2.00 |
| | Benzene | 309 | 0.05 | 0.31 | 0.00 | 0.00 | 0.00 | 0.00 | 3.00 |
| | Chloroform | 13481 | 0.05 | 0.32 | 0.00 | 0.00 | 0.00 | 0.00 | 6.00 |
| | DMSO | 1415 | 0.02 | 0.15 | 0.00 | 0.00 | 0.00 | 0.00 | 2.00 |
| | Methanol | 2060 | 0.02 | 0.16 | 0.00 | 0.00 | 0.00 | 0.00 | 4.00 |
| | Pyridine | 2496 | 0.00 | 0.07 | 0.00 | 0.00 | 0.00 | 0.00 | 1.00 |
| HBA | Acetone | 822 | 6.18 | 2.94 | 1.00 | 4.00 | 6.00 | 7.00 | 26.00 |
| | Benzene | 309 | 2.94 | 2.52 | 0.00 | 1.00 | 3.00 | 4.00 | 12.00 |
| | Chloroform | 13481 | 5.12 | 3.26 | 0.00 | 3.00 | 4.00 | 7.00 | 35.00 |
| | DMSO | 1415 | 7.88 | 4.38 | 1.00 | 5.00 | 7.00 | 10.00 | 33.00 |
| | Methanol | 2060 | 8.03 | 5.01 | 1.00 | 4.00 | 7.00 | 11.00 | 36.00 |
| | Pyridine | 2496 | 8.91 | 5.68 | 0.00 | 5.00 | 8.00 | 11.00 | 34.00 |

| | | | | | | | | | |
|---|---|---|---|---|---|---|---|---|---|
| HBD | Acetone | 822 | 2.97 | 1.82 | 0.00 | 2.00 | 3.00 | 4.00 | 15.00 |
| | Benzene | 309 | 0.78 | 0.89 | 0.00 | 0.00 | 1.00 | 1.00 | 6.00 |
| | Chloroform | 13481 | 1.34 | 1.19 | 0.00 | 0.00 | 1.00 | 2.00 | 14.00 |
| | DMSO | 1415 | 3.69 | 2.88 | 0.00 | 2.00 | 3.00 | 5.00 | 18.00 |
| | Methanol | 2060 | 4.17 | 2.97 | 0.00 | 2.00 | 3.00 | 6.00 | 19.00 |
| | Pyridine | 2496 | 4.85 | 3.54 | 0.00 | 2.00 | 4.00 | 7.00 | 20.00 |
| Number of heavy atoms | Acetone | 822 | 28.47 | 8.82 | 11.00 | 22.00 | 27.00 | 32.00 | 76.00 |
| | Benzene | 309 | 22.65 | 8.17 | 10.00 | 16.00 | 20.00 | 29.00 | 50.00 |
| | Chloroform | 13481 | 29.22 | 9.60 | 5.00 | 22.00 | 28.00 | 35.00 | 112.00 |
| | DMSO | 1415 | 29.81 | 10.38 | 10.00 | 23.00 | 27.00 | 34.00 | 85.00 |
| | Methanol | 2060 | 33.28 | 13.23 | 9.00 | 24.00 | 31.00 | 40.00 | 105.00 |
| | Pyridine | 2496 | 39.96 | 15.40 | 9.00 | 29.75 | 36.00 | 46.00 | 109.00 |
| Number of heteroatoms | Acetone | 822 | 6.25 | 2.91 | 1.00 | 5.00 | 6.00 | 7.00 | 26.00 |
| | Benzene | 309 | 3.02 | 2.57 | 0.00 | 1.00 | 3.00 | 5.00 | 12.00 |
| | Chloroform | 13481 | 5.27 | 3.24 | 0.00 | 3.00 | 5.00 | 7.00 | 35.00 |
| | DMSO | 1415 | 8.11 | 4.46 | 1.00 | 5.00 | 7.00 | 10.00 | 35.00 |
| | Methanol | 2060 | 8.32 | 5.06 | 1.00 | 4.00 | 7.00 | 11.00 | 36.00 |
| | Pyridine | 2496 | 9.22 | 5.62 | 0.00 | 5.00 | 8.00 | 11.00 | 34.00 |
| Number of | Acetone | 822 | 0.79 | 0.86 | 0.00 | 0.00 | 1.00 | 1.00 | 5.00 |

| | | | | | | | | | |
|---|---|---|---|---|---|---|---|---|---|
| alicyclic rings with heteroatoms | Benzene | 309 | 0.59 | 0.95 | 0.00 | 0.00 | 0.00 | 1.00 | 5.00 |
| | Chloroform | 13481 | 0.76 | 1.00 | 0.00 | 0.00 | 0.00 | 1.00 | 9.00 |
| | DMSO | 1415 | 0.92 | 1.06 | 0.00 | 0.00 | 1.00 | 1.00 | 6.00 |
| | Methanol | 2060 | 1.19 | 1.39 | 0.00 | 0.00 | 1.00 | 2.00 | 13.00 |
| | Pyridine | 2496 | 1.51 | 1.55 | 0.00 | 0.00 | 1.00 | 2.00 | 12.00 |
| Number of aromatic rings with heteroatoms | Acetone | 822 | 0.40 | 0.51 | 0.00 | 0.00 | 0.00 | 1.00 | 2.00 |
| | Benzene | 309 | 0.16 | 0.36 | 0.00 | 0.00 | 0.00 | 0.00 | 1.00 |
| | Chloroform | 13481 | 0.21 | 0.45 | 0.00 | 0.00 | 0.00 | 0.00 | 4.00 |
| | DMSO | 1415 | 0.60 | 0.55 | 0.00 | 0.00 | 1.00 | 1.00 | 4.00 |
| | Methanol | 2060 | 0.28 | 0.49 | 0.00 | 0.00 | 0.00 | 1.00 | 4.00 |
| | Pyridine | 2496 | 0.09 | 0.30 | 0.00 | 0.00 | 0.00 | 0.00 | 2.00 |
| Number of iodine atoms | Acetone | 822 | 0.00 | 0.00 | 0.00 | 0.00 | 0.00 | 0.00 | 0.00 |
| | Benzene | 309 | 0.00 | 0.00 | 0.00 | 0.00 | 0.00 | 0.00 | 0.00 |
| | Chloroform | 13481 | 0.00 | 0.04 | 0.00 | 0.00 | 0.00 | 0.00 | 2.00 |
| | DMSO | 1415 | 0.00 | 0.05 | 0.00 | 0.00 | 0.00 | 0.00 | 1.00 |
| | Methanol | 2060 | 0.00 | 0.03 | 0.00 | 0.00 | 0.00 | 0.00 | 1.00 |
| | Pyridine | 2496 | 0.00 | 0.00 | 0.00 | 0.00 | 0.00 | 0.00 | 0.00 |
| CLogP | Acetone | 822 | 3.44 | 2.02 | -3.20 | 2.08 | 3.36 | 4.78 | 11.66 |
| | Benzene | 309 | 4.22 | 1.32 | 1.02 | 3.59 | 4.11 | 4.75 | 13.05 |

| | | | | | | | | | |
|---|---|---|---|---|---|---|---|---|---|
| | Chloroform | 13481 | 4.17 | 2.08 | -3.77 | 2.79 | 3.92 | 5.24 | 15.73 |
| | DMSO | 1415 | 2.01 | 2.22 | -7.96 | 0.95 | 2.37 | 3.22 | 10.38 |
| | Methanol | 2060 | 2.02 | 2.61 | -8.04 | 0.21 | 1.98 | 3.64 | 10.63 |
| | Pyridine | 2496 | 2.38 | 2.75 | -6.58 | 0.58 | 2.25 | 4.27 | 13.68 |
| MW | Acetone | 822 | 393.17 | 122.48 | 152.15 | 308.34 | 378.45 | 440.49 | 1081.26 |
| | Benzene | 309 | 315.73 | 115.85 | 136.24 | 220.36 | 284.44 | 416.51 | 702.97 |
| | Chloroform | 13481 | 407.69 | 134.34 | 66.10 | 312.36 | 386.53 | 482.70 | 1597.71 |
| | DMSO | 1415 | 416.11 | 149.97 | 135.13 | 310.84 | 382.30 | 485.54 | 1225.03 |
| | Methanol | 2060 | 468.60 | 189.63 | 138.17 | 333.83 | 430.50 | 562.53 | 1487.38 |
| | Pyridine | 2496 | 564.20 | 223.24 | 132.16 | 418.53 | 500.63 | 656.75 | 1557.86 |
| Number of nitrogen atoms | Acetone | 822 | 0.02 | 0.15 | 0.00 | 0.00 | 0.00 | 0.00 | 2.00 |
| | Benzene | 309 | 0.08 | 0.45 | 0.00 | 0.00 | 0.00 | 0.00 | 4.00 |
| | Chloroform | 13481 | 0.06 | 0.30 | 0.00 | 0.00 | 0.00 | 0.00 | 5.00 |
| | DMSO | 1415 | 0.10 | 0.51 | 0.00 | 0.00 | 0.00 | 0.00 | 5.00 |
| | Methanol | 2060 | 0.09 | 0.41 | 0.00 | 0.00 | 0.00 | 0.00 | 5.00 |
| | Pyridine | 2496 | 0.02 | 0.17 | 0.00 | 0.00 | 0.00 | 0.00 | 4.00 |
| Number of oxygen atoms | Acetone | 822 | 6.21 | 2.92 | 1.00 | 4.00 | 6.00 | 7.00 | 26.00 |
| | Benzene | 309 | 2.89 | 2.51 | 0.00 | 1.00 | 2.00 | 4.00 | 12.00 |
| | Chloroform | 13481 | 5.15 | 3.22 | 0.00 | 3.00 | 4.00 | 7.00 | 35.00 |

| | | | | | | | | | |
|---|---|---|---|---|---|---|---|---|---|
| | DMSO | 1415 | 7.94 | 4.42 | 1.00 | 5.00 | 7.00 | 10.00 | 35.00 |
| | Methanol | 2060 | 8.17 | 5.06 | 0.00 | 4.00 | 7.00 | 11.00 | 36.00 |
| | Pyridine | 2496 | 9.19 | 5.62 | 0.00 | 5.00 | 8.00 | 11.00 | 34.00 |
| Number of phosphorus atoms | Acetone | 822 | 0.00 | 0.00 | 0.00 | 0.00 | 0.00 | 0.00 | 0.00 |
| | Benzene | 309 | 0.00 | 0.00 | 0.00 | 0.00 | 0.00 | 0.00 | 0.00 |
| | Chloroform | 13481 | 0.00 | 0.00 | 0.00 | 0.00 | 0.00 | 0.00 | 0.00 |
| | DMSO | 1415 | 0.00 | 0.00 | 0.00 | 0.00 | 0.00 | 0.00 | 0.00 |
| | Methanol | 2060 | 0.00 | 0.00 | 0.00 | 0.00 | 0.00 | 0.00 | 0.00 |
| | Pyridine | 2496 | 0.00 | 0.00 | 0.00 | 0.00 | 0.00 | 0.00 | 0.00 |
| Number of ring systems | Acetone | 822 | 3.63 | 1.32 | 0.00 | 3.00 | 3.00 | 4.00 | 10.00 |
| | Benzene | 309 | 2.82 | 1.16 | 0.00 | 2.00 | 3.00 | 3.00 | 6.00 |
| | Chloroform | 13481 | 3.68 | 1.57 | 0.00 | 3.00 | 3.00 | 5.00 | 13.00 |
| | DMSO | 1415 | 3.87 | 1.43 | 0.00 | 3.00 | 3.00 | 5.00 | 12.00 |
| | Methanol | 2060 | 4.06 | 1.82 | 0.00 | 3.00 | 4.00 | 5.00 | 13.00 |
| | Pyridine | 2496 | 5.41 | 1.91 | 0.00 | 4.00 | 5.00 | 7.00 | 17.00 |
| Number of rotable bonds | Acetone | 822 | 3.57 | 2.79 | 0.00 | 1.00 | 3.00 | 5.00 | 18.00 |
| | Benzene | 309 | 2.68 | 2.89 | 0.00 | 1.00 | 2.00 | 4.00 | 18.00 |
| | Chloroform | 13481 | 3.69 | 3.31 | 0.00 | 1.00 | 3.00 | 5.00 | 41.00 |
| | DMSO | 1415 | 3.79 | 3.02 | 0.00 | 2.00 | 3.00 | 5.00 | 22.00 |

| | | | | | | | | | |
|---|---|---|---|---|---|---|---|---|---|
| | Methanol | 2060 | 5.19 | 4.10 | 0.00 | 2.00 | 4.00 | 7.00 | 51.00 |
| | Pyridine | 2496 | 4.86 | 4.10 | 0.00 | 2.00 | 4.00 | 7.00 | 38.00 |
| TPSA | Acetone | 822 | 101.78 | 46.21 | 9.23 | 73.83 | 96.22 | 116.45 | 444.18 |
| | Benzene | 309 | 44.07 | 36.10 | 0.00 | 17.07 | 38.83 | 66.76 | 168.80 |
| | Chloroform | 13481 | 77.75 | 44.25 | 0.00 | 46.53 | 69.67 | 99.13 | 439.13 |
| | DMSO | 1415 | 128.27 | 71.95 | 9.23 | 79.90 | 107.36 | 155.89 | 548.48 |
| | Methanol | 2060 | 134.15 | 77.74 | 9.23 | 77.51 | 113.29 | 177.49 | 557.71 |
| | Pyridine | 2496 | 150.40 | 87.43 | 0.00 | 87.09 | 124.29 | 184.12 | 538.50 |

[a] std: standard deviation.
[b] min: minimum value.
[c] Q1: value under which 25% of data points are found in increasing order.
[d] Q3: value under which 75% of data points are found in increasing order.
[e] max: maximum value.

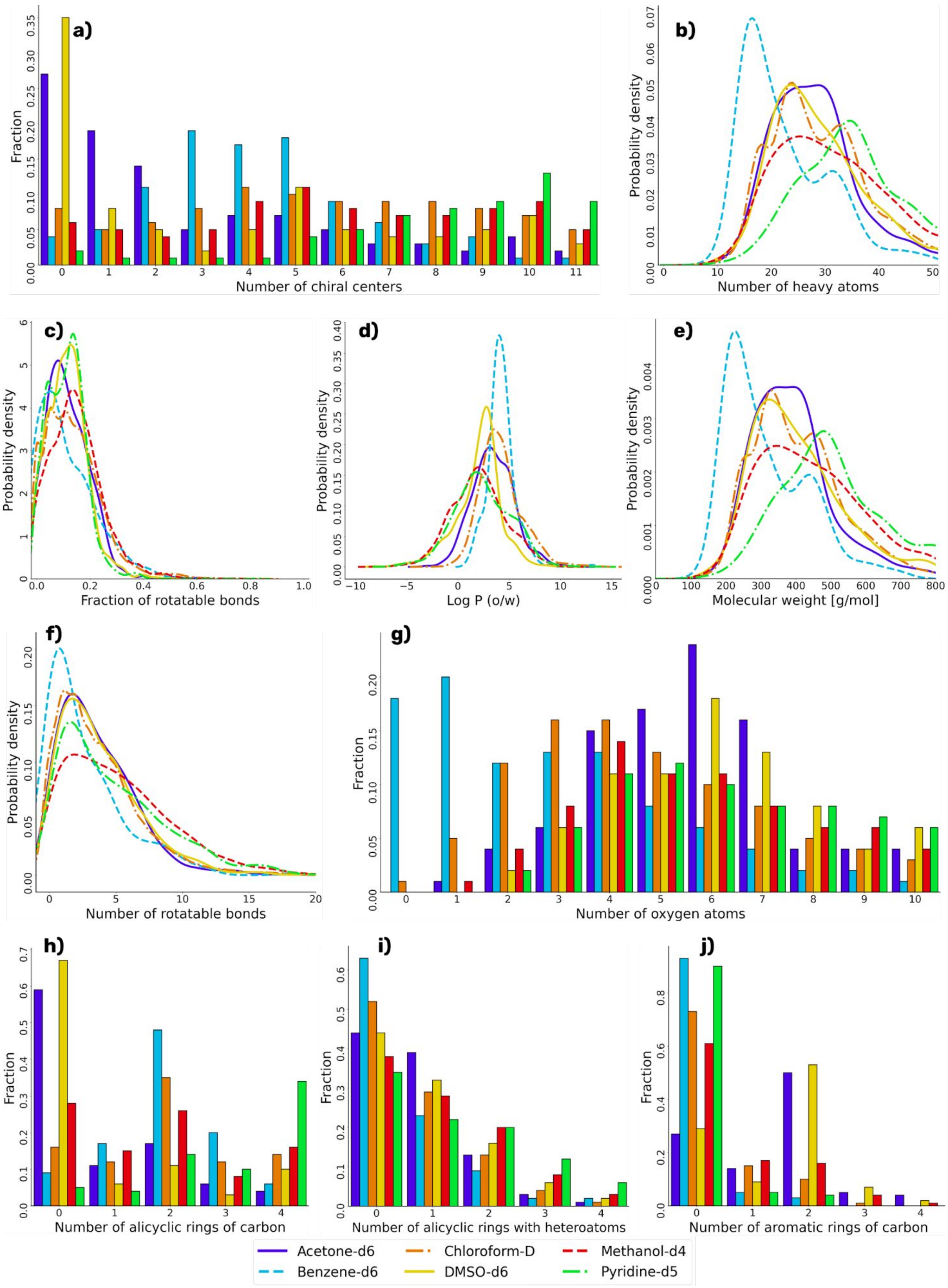


**Figure S3.** Distribution of physicochemical properties and constitutional descriptors of interest computed for NAPROC-13 among their solubility categories.

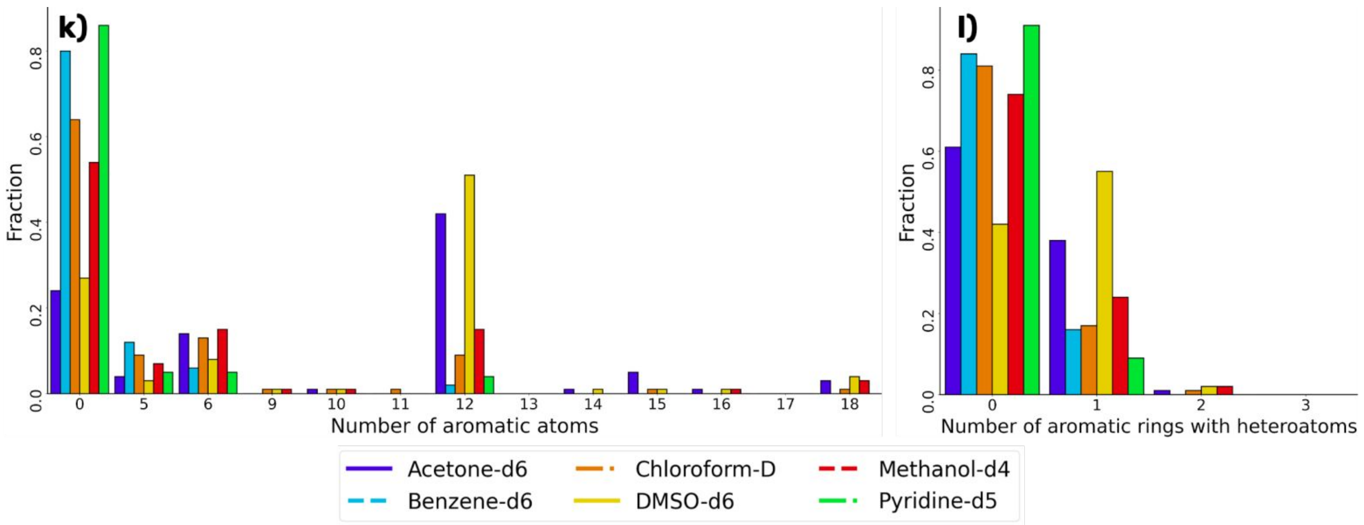


**Figure S3 (cont).** Distribution of physicochemical properties and constitutional descriptors of interest computed for NAPROC-13 among their solubility categories.

**Table S3.** Descriptive statistics of pairwise similarity distribution computed for NPs in NAPROC-13 and UNPDA, and FDA-approved drugs.[d]

| Fingerprint | data set | number of pairwise comparisons | mean | std[a] | Q1[b] | median | Q3[c] |
|---|---|---|---|---|---|---|---|
| MACCS keys 166 bits | NAPROC-13 | 12497500 | 0.540 | 0.139 | 0.446 | 0.549 | 0.640 |
| | UNPD-A | 12497500 | 0.352 | 0.172 | 0.226 | 0.345 | 0.467 |
| | FDA | 2699326 | 0.300 | 0.144 | 0.200 | 0.295 | 0.395 |
| ECFP4 1024 bits | NAPROC-13 | 12497500 | 0.140 | 0.052 | 0.107 | 0.135 | 0.167 |
| | UNPD-A | 12497500 | 0.099 | 0.056 | 0.060 | 0.091 | 0.127 |
| | FDA | 2699326 | 0.096 | 0.051 | 0.065 | 0.094 | 0.124 |
| ECFP6 1024 bits | NAPROC-13 | 12497500 | 0.115 | 0.039 | 0.090 | 0.112 | 0.135 |
| | UNPD-A | 12497500 | 0.082 | 0.043 | 0.053 | 0.078 | 0.105 |
| | FDA | 2699326 | 0.081 | 0.042 | 0.056 | 0.080 | 0.104 |
| MAP4 1024 bits | NAPROC-13 | 12497500 | 0.019 | 0.024 | 0.003 | 0.012 | 0.026 |
| | UNPD-A | 12497500 | 0.010 | 0.019 | 0.000 | 0.002 | 0.012 |
| | FDA | 2699326 | 0.008 | 0.019 | 0.000 | 0.003 | 0.010 |

[a] std: standard deviation.

[b] Q1: value under which 25% of data points are found in increasing order.

[c] Q3: value under which 75% of data points are found in increasing order.

[d] Minimum and maximum values were 0, and 1 respectively for all data sets and all different representations.

**Table S4.** Descriptive statistics of Natural Product-Likeness scores computed for NPs in NAPROC-13, UNPD-A, and FDA-approved drugs.

| data set | NAPROC-13 | UNPD-A | FDA |
|---|---|---|---|
| count | 21250 | 14994 | 2324 |
| mean | 2.437 | 1.513 | 0.019 |
| std[a] | 0.765 | 1.054 | 1.079 |
| min[b] | -1.761 | -2.150 | -2.501 |
| Q1[c] | 1.985 | 0.748 | -0.786 |
| median | 2.575 | 1.505 | -0.095 |
| Q3[d] | 3.007 | 2.322 | 0.626 |
| max[e] | 4.086 | 4.075 | 3.928 |

[a] std: standard deviation.

[b] min: minimum value.

[c] Q1: value under which 25% of data points are found in increasing order.

[d] Q3: value under which 75% of data points are found in increasing order.

[e] max: maximum value.

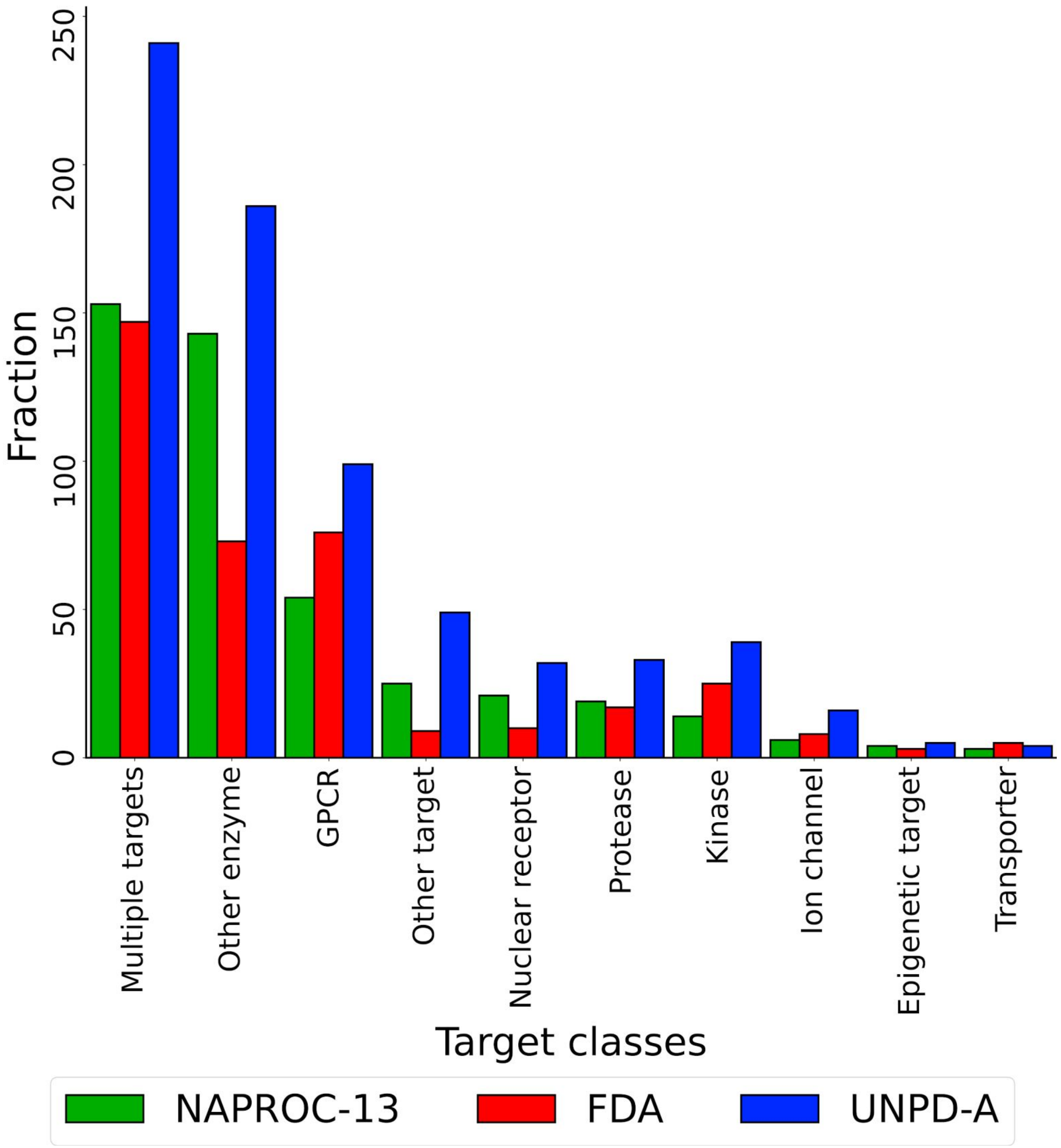


**Figure S4.** Distribution of the "magic-ring systems" in NAPROC-13, FDA-approved drugs, and UNPD-A, among the families of biological targets predicted to be most probable to have biological activity. The most common category was the "not known" target, with 728 ring systems in NAPROC-13, 33 in FDA-approved drugs, and 570 in UNPD-A.

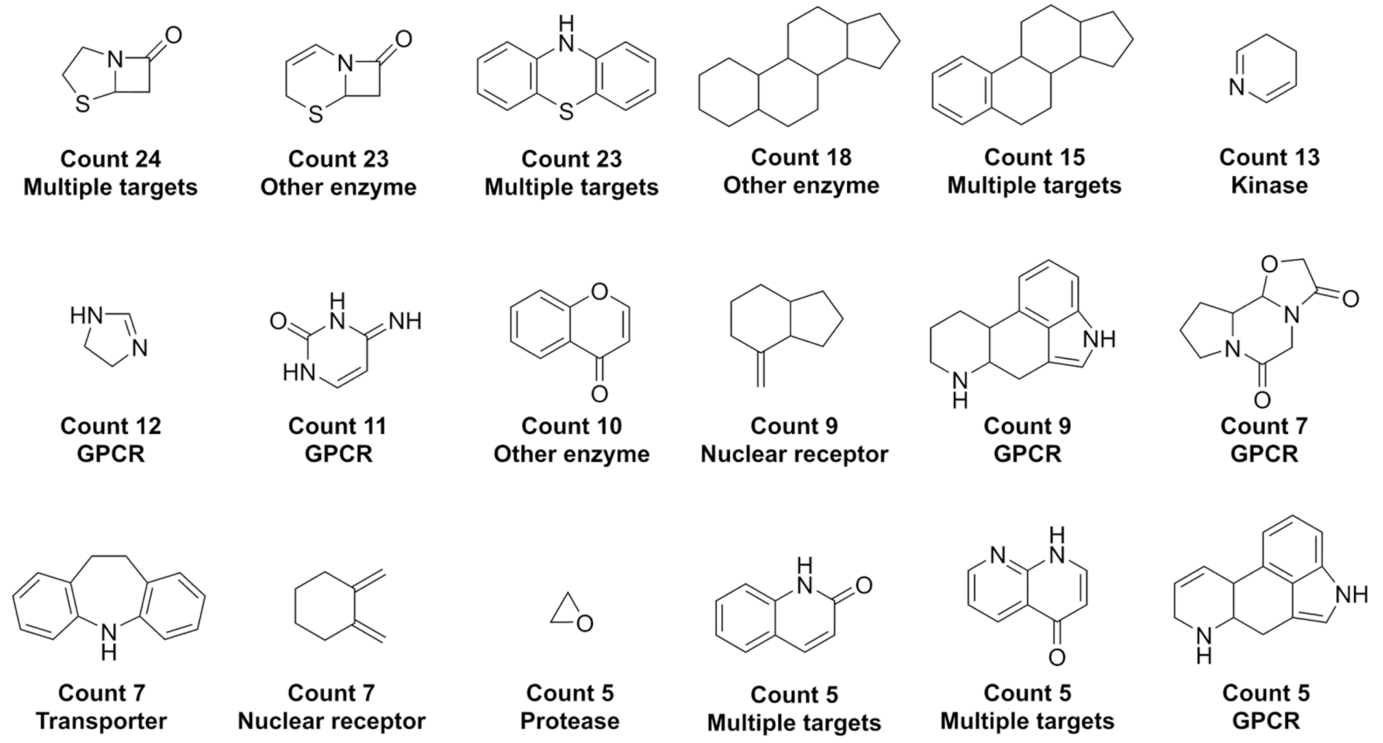


**Figure S5.** Most frequent potentially bioactive ring systems in FDA-approved drugs ("magic rings").

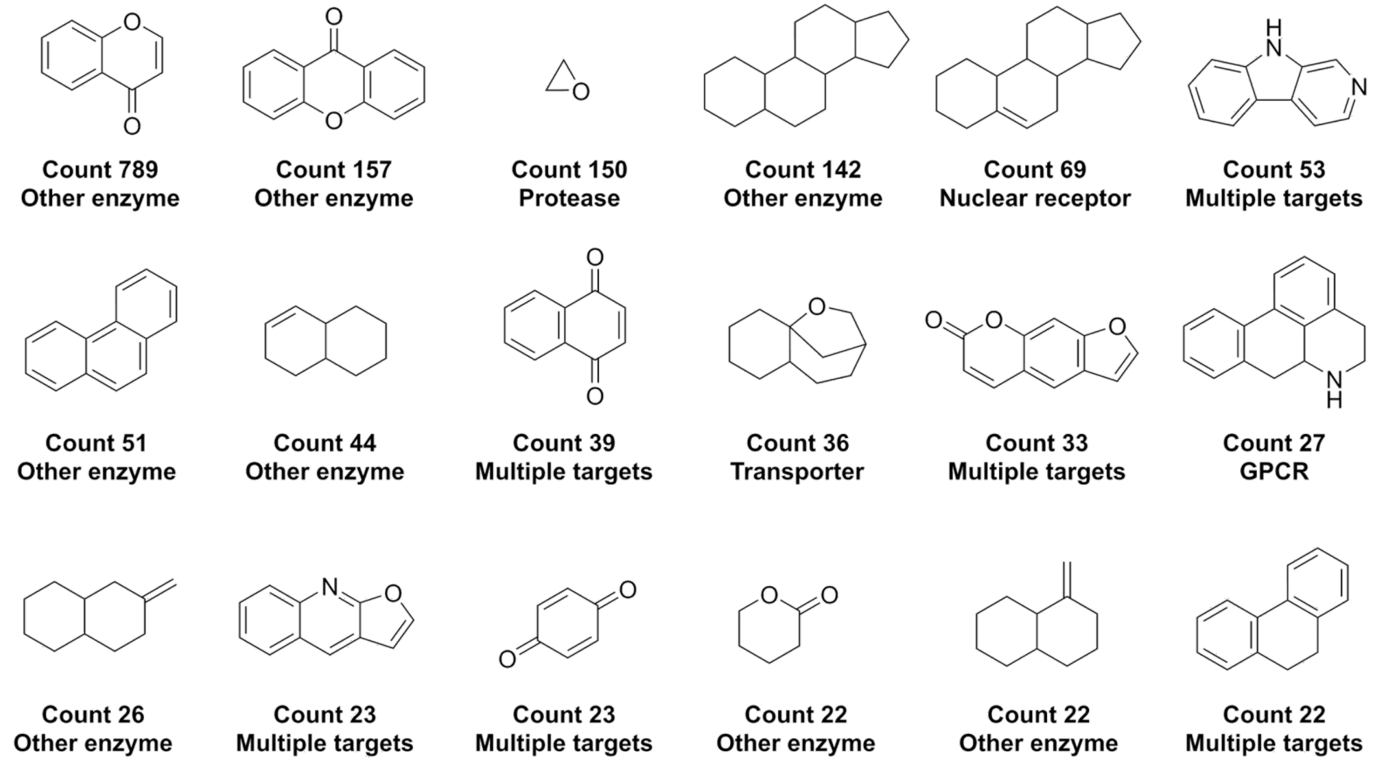


**Figure S6.** Most frequent potentially bioactive ring systems in UNPD-A ("magic rings").
